# Weighted Gene Co-Expression Analyses Point to Long Non-Coding RNA Hub Genes at Different *Schistosoma mansoni* Life-Cycle Stages

**DOI:** 10.3389/fgene.2019.00823

**Published:** 2019-09-12

**Authors:** Lucas F. Maciel, David A. Morales-Vicente, Gilbert O. Silveira, Raphael O. Ribeiro, Giovanna G. O. Olberg, David S. Pires, Murilo S. Amaral, Sergio Verjovski-Almeida

**Affiliations:** ^1^Laboratório de Expressão Gênica em Eucariotos, Instituto Butantan, São Paulo, Brazil; ^2^Programa Interunidades em Bioinformática, Instituto de Matemática e Estatística, Universidade de São Paulo, São Paulo, Brazil; ^3^Departamento de Bioquímica, Instituto de Química, Universidade de São Paulo, São Paulo, Brazil

**Keywords:** parasitology, RNA-seq, single-cell sequencing data, *Schistosoma mansoni*, long non-coding RNAs, weighted genes co-expression network analysis

## Abstract

Long non-coding RNAs (lncRNAs) (>200 nt) are expressed at levels lower than those of the protein-coding mRNAs, and in all eukaryotic model species where they have been characterized, they are transcribed from thousands of different genomic *loci*. In humans, some four dozen lncRNAs have been studied in detail, and they have been shown to play important roles in transcriptional regulation, acting in conjunction with transcription factors and epigenetic marks to modulate the tissue-type specific programs of transcriptional gene activation and repression. In *Schistosoma mansoni*, around 10,000 lncRNAs have been identified in previous works. However, the limited number of RNA-sequencing (RNA-seq) libraries that had been previously assessed, together with the use of old and incomplete versions of the *S. mansoni* genome and protein-coding transcriptome annotations, have hampered the identification of all lncRNAs expressed in the parasite. Here we have used 633 publicly available *S. mansoni* RNA-seq libraries from whole worms at different stages (n = 121), from isolated tissues (n = 24), from cell-populations (n = 81), and from single-cells (n = 407). We have assembled a set of 16,583 lncRNA transcripts originated from 10,024 genes, of which 11,022 are novel *S. mansoni* lncRNA transcripts, whereas the remaining 5,561 transcripts comprise 120 lncRNAs that are identical to and 5,441 lncRNAs that have gene overlap with *S. mansoni* lncRNAs already reported in previous works. Most importantly, our more stringent assembly and filtering pipeline has identified and removed a set of 4,293 lncRNA transcripts from previous publications that were in fact derived from partially processed mRNAs with intron retention. We have used weighted gene co-expression network analyses and identified 15 different gene co-expression modules. Each parasite life-cycle stage has at least one highly correlated gene co-expression module, and each module is comprised of hundreds to thousands lncRNAs and mRNAs having correlated co-expression patterns at different stages. Inspection of the top most highly connected genes within the modules’ networks has shown that different lncRNAs are hub genes at different life-cycle stages, being among the most promising candidate lncRNAs to be further explored for functional characterization.

## Introduction

Schistosomiasis is a neglected tropical disease, caused by flatworms from the genus *Schistosoma*, with estimates of more than 250 million infected people worldwide and responsible for 200 thousand deaths annually at the Sub-Saharan Africa (Who, 2015). *Schistosoma mansoni*, prevalent in Africa and Latin America, is one of the three main species related to human infections (Cdc, 2018). In America, it is estimated that 1 to 3 million people are infected by *S. mansoni* and over 25 million live in risk areas, being Brazil and Venezuela the most affected (Zoni et al., 2016). The prevalence of this disease is correlated to social–economic and environmental factors (Gomes Casavechia et al., 2018).

This parasite has a very complex life-cycle comprised of several developmental stages, with a freshwater snail intermediate-host and a final mammalian host (Basch, 1976). Recently, it has been shown that epigenetic changes are required for life-cycle progression (Roquis et al., 2018). However, little is known about the genes and molecules that drive this process through the life-cycle stages of *S. mansoni*. A better understanding of the gene expression regulation mechanisms and of their components may lead to new therapeutic targets (Batugedara et al., 2017), and one key element could be the long non-coding RNAs (lncRNAs) (Blokhin et al., 2018).

LncRNAs are defined as transcripts longer than 200 nucleotides, without apparent protein-coding potential (Cao et al., 2018). The term “apparent” is included because it is already known that some lncRNAs actually have dual function roles, being functional both as lncRNAs and through peptides shorter than 100 amino acids that they encode (Nam et al., 2016; Choi et al., 2018). In mammalians, lncRNAs regulate gene expression through different mechanisms (Bhat et al., 2016), including mediating epigenetic modifications (Hanly et al., 2018), and were shown to be important in vital processes, such as cell cycle regulation (Kitagawa et al., 2013), pluripotency maintenance (Rosa and Ballarino, 2016), and reproduction (Golicz et al., 2018).

In *S. mansoni*, the expression of lncRNAs at different life-cycle stages was first detected by our group in 2011 using microarrays (Oliveira et al., 2011). Subsequently, large-scale identification of *S. mansoni* lncRNAs has been reported in three studies from our group and from others that analyzed high-throughput RNA-sequencing (RNA-seq) data (Vasconcelos et al., 2017; Liao et al., 2018; Oliveira et al., 2018), but each of them has used a the limited number of data sets (from 4 to 88 RNA-seq libraries). Because each work used different mapping tools and parameters (Vasconcelos et al., 2017; Liao et al., 2018; Oliveira et al., 2018), and given that Liao et al. (2018) did not compare their lncRNAs with the previously published ones, part of the lncRNAs are redundant among the three reports. In addition, the lncRNAs were annotated against the old version 5.2 of the genome and protein-coding transcriptome (Protasio et al., 2012); as a result, a set of transcripts that were previously annotated as lncRNAs (Vasconcelos et al., 2017; Liao et al., 2018; Oliveira et al., 2018), seem now to represent partially processed pre-mRNAs arising from novel protein-coding genes annotated in the new version 7.1 of the transcriptome (https://parasite.wormbase.org/Schistosoma_mansoni_prjea36577/); these transcripts were previously annotated as having no coding potential due to intron retention, as exemplified in **Supplementary Figure S1**. Besides, these three works used expression data from whole parasites, while it is known from other species that lncRNAs have tissue- and cell-specific expression (Wu et al., 2016; Credendino et al., 2017).

The aim of the present work is to identify and annotate a robust and more complete set of lncRNAs that agrees with the most updated transcriptome annotation, and to analyze RNA-seq data sets still non-annotated for the presence of lncRNAs—e.g., gonads (Lu et al., 2016) and single-cell (Tarashansky et al., 2018; Wang et al., 2018) RNA-seq libraries. The goal is to provide a foundation that will enable future studies on the role of lncRNAs in *S. mansoni* biology, which could eventually identify potential new therapeutic targets.

## Materials and Methods

### Transcripts Reconstruction

To identify new lncRNAs, 633 publicly available RNA-seq libraries from whole worms at different stages (miracidia, n = 1; sporocysts, n = 1; cercariae, n = 8; schistosomula, n = 11; juveniles, n = 9; adult males, n = 34; adult females, n = 37; and mixed adults, n = 20), from tissues (testes, n = 6; ovaries, n = 5; posterior somatic tissues, n = 3; heads, n = 5; and tails, n = 5), from cell populations (n = 81) and from single cells (from juveniles, n = 370 and mother sporocysts stem cells, n = 37) were downloaded from the SRA and ENA databases (**Supplementary Table S1**). The only whole-worm stage that was not included was eggs, because there is a single RNA-seq library available in the public domain (Anderson et al., 2016), which has only 252,000 egg reads, an amount that is fourfold lower than the minimum number of reads per library in the other whole-worm libraries that we used (namely 1 million good quality reads), being a too-low coverage for an unbiased detection of stage- or tissue-specific lncRNAs in complex organisms (Sims et al., 2014). The new versions of the genome (v 7) and transcriptome (v 7.1), which were used as reference in this study, were downloaded from the WormBase ParaSite resource (Howe et al., 2017) at https://parasite.wormbase.org/Schistosoma_mansoni_prjea36577/. 

Quality control was done with fastp v 0.19.4 (Chen et al., 2018) (default parameters), removing adapters and low-quality reads. The reads in each library were then mapped against the genome with STAR v 2.6.1c in a two-pass mode, with parameters indicated by STAR’s manual as the best ones to identify new splicing sites and transcripts (Dobin et al., 2013). RSeQC v 2.6.5 (Wang et al., 2012) was used to identify RNA-Seq library strandedness to be used in transcripts reconstruction and expression levels quantification. For each library, multi mapped reads were removed with Samtools v 1.3 (Li et al., 2009) and uniquely mapped reads were used for transcript reconstruction with Scallop v 10.2 (–min_mapping_quality 255 -min_splice_boundary_hits 2) (Shao and Kingsford, 2017). A new splicing site should be confirmed at least by two reads to be considered. A consensus transcriptome from all libraries was built using TACO v 0.7.3 (–filter-min-length 200 -isoform-frac 0.05), an algorithm that reconstructs the consensus transcriptome from a collection of individual assemblies (Niknafs et al., 2016). As described by Niknafs et al. (2016), TACO employs change point detection to break apart complex loci and correctly delineate transcript start and end sites and a dynamic programming approach to assemble transcripts from a network of splicing patterns (Niknafs et al., 2016).

### LncRNAs Classification

In the consensus transcriptome, transcripts shorter than 200 nt, monoexonic or with exon-exon overlap with protein-coding genes from the same genomic strand were removed from the set. The coding potential of the remaining transcripts was evaluated by means of the FEELnc tool v 0.1.1 (Wucher et al., 2017) with shuffle mode, which uses a random forest machine-learning algorithm and classifies these transcripts into lncRNAs or protein-coding genes, and also by CPC2 v 0.1 (Yang et al., 2017), which classifies through a support vector machine model using four intrinsic features. Only transcripts classified as lncRNAs by both tools were kept. ORFfinder v 0.4.3 (https://www.ncbi.nlm.nih.gov/orffinder/) was used to extract the putative longest open reading frames (ORFs); these putative peptides were then submitted to orthology-based annotation with eggNOG-mapper webtool (HMMER mapping mode) (Huerta-Cepas et al., 2017). Transcripts with no hits against the eukaryote eggNOG database were then considered as lncRNAs. If any transcript isoform was classified as a protein-coding mRNA at any step, all transcripts mapping to the same genomic *locus* were removed to avoid eventual pre-mRNAs. After this final step, a lncRNAs GTF file was created.

### Histone Marks

To identify histone H3 lysine 4 trimethylation (H3K4me3) and H3 lysine 27 trimethylation (H3K27me3) marks near the transcription start site (TSS) of lncRNAs, we used 12 libraries of Chromatin Immunoprecipitation Sequencing (ChIP-Seq) data generated by Roquis et al. (2018) for cercariae, schistosomula, and adults (**Supplementary Table S1**), which had more than 90% overall mapping rate. The reads were downloaded from the SRA database and mapped against the genome v 7 with Bowtie2 v 2.3.4.3 (Langmead and Salzberg, 2012) (parameters end-to-end, -sensitive, -gbar 4). Because there are no input data sets publicly available in the SRA database for the Roquis et al. (2018) paper, we were not able to exactly reproduce the pipeline that was described in the Methods section of that paper, which used the input as a reference for peak calling. Instead, we used HOMER v 4.10 (Heinz et al., 2010) for removing multi-mapped and duplicated reads and for significant peak calling as described by Anderson et al. (2016), an approach also used by Vasconcelos et al. (2017) in the first large-scale annotation of lncRNAs in *S. mansoni*. The number of reads in the peak should be at least fourfold higher than in the peaks of the surrounding 10-kb area and the Poisson p-value threshold cutoff was 0.0001. The lncRNAs with significant histone mark peaks within 1-kb distance upstream and downstream from their TSS were annotated. The lncRNAs with overlapping marks are shown with an intersection diagram that was plotted using the UpSetR tool v 1.3.3 (Lex et al., 2014). The Venn diagram tool at http://bioinformatics.psb.ugent.be/beg/tools/venn-diagrams was used for generating the lists of lncRNA genes belonging to each intersection set.

### Co-Expression Networks

The lncRNAs GTF file was then added to the *S. mansoni* public protein-coding transcriptome version 7.1 GTF file, and the resulting protein-coding + lncRNAs GTF was used as the reference together with the genome sequence v.7 for mapping the reads of each RNA-seq library under study, again using the STAR tool, now in the one pass mode, followed by gene expression quantification with RSEM v 1.3 (Li and Dewey, 2011). Weighted gene co-expression network analyses v 1.68 (WGCNA) (Langfelder and Horvath, 2008) were then performed to identify modules related to the life-cycle stages and tissues of the organism. For this purpose, only libraries from whole worms or from tissues with more than 50% of the reads uniquely mapped were used. To reduce noise, only transcripts with expression greater than 1 transcript per million (TPM) in at least half of the libraries in one or more stages/tissues were considered. Expression levels were measured in log space with a pseudocount of 1 (log2 (TPM+1)), and we set the transcript expression to zero when log2 (TPM+1) <1. For the construction of the adjacency matrix, the power adjacency function for signed networks was applied with the soft-thresholding beta parameter equal to 14, which resulted in a scale-free topology model fit index (*R* ^2^ = 0.935). The adjacency matrix was then converted to the Topological Overlap Matrix (TOM) and the dissimilarity TOM (1 − TOM) was calculated (Langfelder and Horvath, 2008).

Correlation between the modules and the stages was calculated based on the Pearson correlation coefficient between the expression levels of the transcripts belonging to each module along the stages, as suggested in the WGCNA tutorial (https://horvath.genetics.ucla.edu/html/CoexpressionNetwork/Rpackages/WGCNA/Tutorials/). As miracidia and sporocysts have only one library each, are closely related stages of development, and were clustered together as an outgroup based on their overall expression patterns (as shown in the Results), we decided to consider both stages together as one group (miracidia/sporocysts) to calculate the correlation and p-values between modules and stages.

The Gene Trait Significance (GS) was calculated based on the correlation of an individual transcript and the trait, which in our case was always the stage of higher absolute Pearson correlation coefficient with the module where the transcript belongs. For example, for a transcript that belongs to the red module (most highly correlated with testes, see Results), the correlation was calculated between the expression of the transcript in the testes libraries and the expression of the transcript in all other non-testes libraries.

### Gene Ontology (GO) Enrichment

Protein-coding genes were submitted to eggNOG-mapper (Huerta-Cepas et al., 2017) for annotation of GO terms. Based on this annotation (available at **Supplementary Table S2**), we performed GO enrichment analyses with BINGO (Maere et al., 2005). For each module, we used a hypergeometric test, the whole annotation as reference set, and FDR ≤ 0.05 was used as the significance threshold.

### Single-Cell Analyses

The expression levels were quantified in single-cell RNA-seq libraries from juveniles’ stem cells (Tarashansky et al., 2018) and mother sporocysts stem cells (Wang et al., 2018) by RSEM. We used Scater v 1.10.1 (Mccarthy et al., 2017) to normalize and identify high-quality single-cell RNA-Seq libraries, i.e., those that have at least 100,000 total counts and at least 1,000 different expressed transcripts, as recommended by Mccarthy et al. (2017); all libraries were classified as high quality.

Next, we used the R package Single-Cell Consensus Clustering (SC3) tool v 1.10.1 (Kiselev et al., 2017), which performs an unsupervised clustering of scRNA-seq data. Based on the clusters identified, we used the plot SC3 markers function to find marker genes based on the mean cluster expression values. These markers are highly expressed in only one of the clusters and indicate the specific expression at the cell level. As described by Kiselev et al. (2017), the area under the receiver operating characteristic (ROC) curve is used to quantify the accuracy of the prediction. A p-value is assigned to each gene by using the Wilcoxon signed rank test. Genes with the area under the ROC curve (AUROC) > 0.85 and with p-value < 0.01 are defined as marker genes.

### Parasite Materials

All parasite materials were from a BH isolate of *S. mansoni* maintained by passage through golden hamster (*Mesocricetus auratus*) and *Biomphalaria glabrata* snails. Eggs were purified from livers of hamsters previously infected with *S. mansoni*, according to Dalton et al. (1997). After purification, eggs were added to 10 ml of distilled water and exposed to a bright light. Supernatant containing hatched miracidia was removed every 30 min for 2 h and replaced by fresh water. The supernatants containing the miracidia were pooled and chilled on ice, and miracidia were then recovered by centrifugation at 15,000*g* for 20 s (Dalton et al., 1997). Supernatant was discarded and miracidia stored in RNAlater (Ambion) until RNA extraction.

Cercariae were collected from snails infected with 10 miracidia each. Thirty-five days after infection, the snails were placed in the dark in water and then illuminated for 2 h to induce shedding. The emerging cercariae were collected by centrifugation, washed with PBS once, and then stored in RNAlater (Ambion) until RNA extraction.

Schistosomula were obtained by mechanical transformation of cercariae and separation of their bodies as previously described (Basch, 1981), with some modifications. Briefly, cercariae were collected as described above and then suspended in 15 ml of M169 medium (Vitrocell, cat number 00464) containing penicillin/streptomycin, amphotericin (Vitrocell, cat number 00148). Mechanical transformation was performed by passing the cercariae 10 times through a 23G needle. To separate schistosomula from the tails, the tail-rich supernatant was decanted and the sedimented bodies resuspended in a further 7 ml of M169 medium. The procedure was repeated until less than 1% of the tails remained. The newly transformed schistosomula were maintained for 24 h in M169 medium (Vitrocell, cat number 00464) supplemented with penicillin/streptomycin, amphotericin, gentamicin (Vitrocell, cat number 00148), 2% fetal bovine serum, 1 μM serotonin, 0.5 μM hypoxanthine, 1 μM hydrocortisone, and 0.2 μM triiodothyronine at 37°C and 5% CO_2_. Schistosomula cultivated for 24 h were collected, washed three times with PBS and stored in RNAlater (Ambion) until RNA extraction.

Adult *S. mansoni* worms were recovered by perfusion of golden hamsters that had been infected with 250 cercariae, 7 weeks previously. Approximately 200 *S. mansoni* (BH strain) adult worm pairs were freshly obtained through the periportal perfusion of hamster, as previously described (Anderson et al., 2016; Vasconcelos et al., 2017). After perfusion, the adult worm pairs were kept for 3 h at 37°C and 5% CO_2_ in Advanced RPMI Medium 1640 (Gibco, 12633-012) supplemented with 10% fetal bovine serum, 12 mM HEPES (4-(2-hydroxyethyl) piperazine-1-ethanesulfonic acid) pH 7.4, and 1% penicillin/streptomycin, amphotericin (Vitrocell, cat number 00148). After 3 h of incubation, the adult worm pairs were collected, washed three times with PBS, and stored in RNAlater (Ambion) until RNA extraction. Before the extraction of RNA from males or females, adult worm pairs were manually separated in RNAlater (Ambion) using tweezers.

### RNA Extraction, Quantification, and Quality Assessment

Total RNA from eggs (E), miracidia (Mi), cercariae (C), and schistosomula (S) was extracted according to Vasconcelos et al. (2017). Briefly, 100,000 eggs, 15,000 miracidia, 25,000 cercariae, or 25,000 schistosomula were ground with glass beads in liquid nitrogen for 5 min. Then, the Qiagen RNeasy Micro Kit (Cat number 74004) was used for RNA extraction and purification according to the manufacturer’s instructions, except for the DNase I treatment, the amount of DNase I was doubled and the time of treatment was increased to 45 min.

Male (M) or female (F) adult worms were first disrupted in Qiagen RLT buffer using glass potters and pestles. RNA from males or females was then extracted and purified using the Qiagen RNeasy Mini Kit (Cat number 74104), according to the manufacturer’s instructions, except for the DNase I treatment, which was the same used for egg, miracidia, cercariae, and schistosomula RNA extraction.

All the RNA samples were quantified using the Qubit RNA HS Assay Kit (Q32852, Thermo Fisher Scientific), and the integrity of RNAs was verified using the Agilent RNA 6000 Pico Kit (5067-1513 Agilent Technologies) in a 2100 Bioanalyzer Instrument (Agilent Technologies). Four biological replicates were assessed for each life cycle stage, except for schistosomula, for which three biological replicates were assessed.

### Reverse Transcription and Quantitative PCR (qPCR) Assays

The reverse transcription (RT) reaction was performed with 200 ng of each total RNA sample using the SuperScript IV First-Strand Synthesis System (18091050; Life Technologies) and random hexamer primers in a 20-μL final volume. The obtained complementary DNAs (cDNAs) were diluted four times in DEPC water, and quantitative PCR was performed using 2.5 μL of each diluted cDNA in a total volume of 10 μL containing 1X LightCycler 480 SYBR Green I Master Mix (04707516001, Roche Diagnostics) and 800 nM of each primer in a LightCycler 480 System (Roche Diagnostics). Primers for selected transcripts (**Supplementary Table S3**) were designed using the Primer 3 tool (http://biotools.umassmed.edu/bioapps/primer3_www.cgi), and each real-time qPCR was run in two technical replicates. The results were analyzed by comparative Ct method (Livak and Schmittgen, 2001). Real-time data were normalized in relation to the level of expression of Smp_090920 and Smp_062630 reference genes.

## Results

### LncRNAs Identification and Annotation

Using 633 publicly available *S. mansoni* RNA-seq libraries from whole worms at different stages, from isolated tissues, from cell-populations, and from single-cells (see Methods), our pipeline assembled a consensus transcriptome comprised of 78,817 transcripts, of which 7,954 were classified as intergenic lncRNAs (lincRNAs), 7,438 as antisense lncRNAs, and 1,191 as sense lncRNAs, totalizing 16,583 lncRNA transcripts originated from 10,024 genes (on average, 1.65 lncRNA isoforms per lncRNA gene); the summary of all six filtering steps in the pipeline is presented in **Table 1**. With the FEELnc lncRNA classification tool (**Table 1**, **step 5**), the most important feature for transcripts classification was the ORF coverage (**Supplementary Figure S2A**), i.e., the fraction of the total length of the transcript that is occupied by the longest predicted ORF. In the FEELnc model training process, an optimal coding probability cutoff (0.348) was identified, which resulted in 0.962 sensitivity and specificity of mRNA classification (**Supplementary Figure S2B**). Analogous information is not provided in the output of the CPC2 classification tool (**Table 1**, **step 5**). Only the lncRNAs classified as such by both prediction tools were retained in the final set (**Table 1**).

**Table 1:** Summary of transcripts removed at each filtering step and the final set of *S. mansoni* lncRNAs.

Pipeline step number	Removed transcripts	Remaining transcripts (Genes)
**1. Total assembled transcripts**		78,817 (42,337)
**2. Remove short transcripts (<200 nt)**	11	78,806
**3. Remove monoexonic transcripts**	27,255	51,551
**4. Remove transcripts that overlap exon-exon with known Sm protein-coding genes**	31,183	20,368
**5. Remove transcripts with coding potential (FEELnc and/or CPC2 tools)**	3,618	16,750
**6. Remove transcripts with hits on eggNOG-Mapper**	167	16,583
**7. Total lncRNAs identified**		16,583 (10,024)
** Long intergenic non-coding RNAs**		7,954
** Antisense long non-coding RNAs**		7,438
** Sense long non-coding RNAs**		1,191

From the total set of 16,583 lncRNAs obtained here, 11,022 are novel *S. mansoni* lncRNAs, whereas the remaining 5,561 transcripts comprise 120 lncRNAs that are identical to previously published ones, and 5,441 lncRNAs that have gene overlap with *S. mansoni* lncRNAs already reported in previous works (Vasconcelos et al., 2017; Liao et al., 2018; Oliveira et al., 2018) (**Supplementary Table S4**). In particular, among the 7,029 lincRNAs previously published ones reported by our group (Vasconcelos et al., 2017), a total of 4,368 transcripts have partial or complete sequence overlap with the lncRNAs obtained here, whereas the remaining 2,661 (37.8%) transcripts previously annotated by Vasconcelos et al. (2017) are no longer in the present updated *S. mansoni* lncRNAs data set.

**Figure 1:**
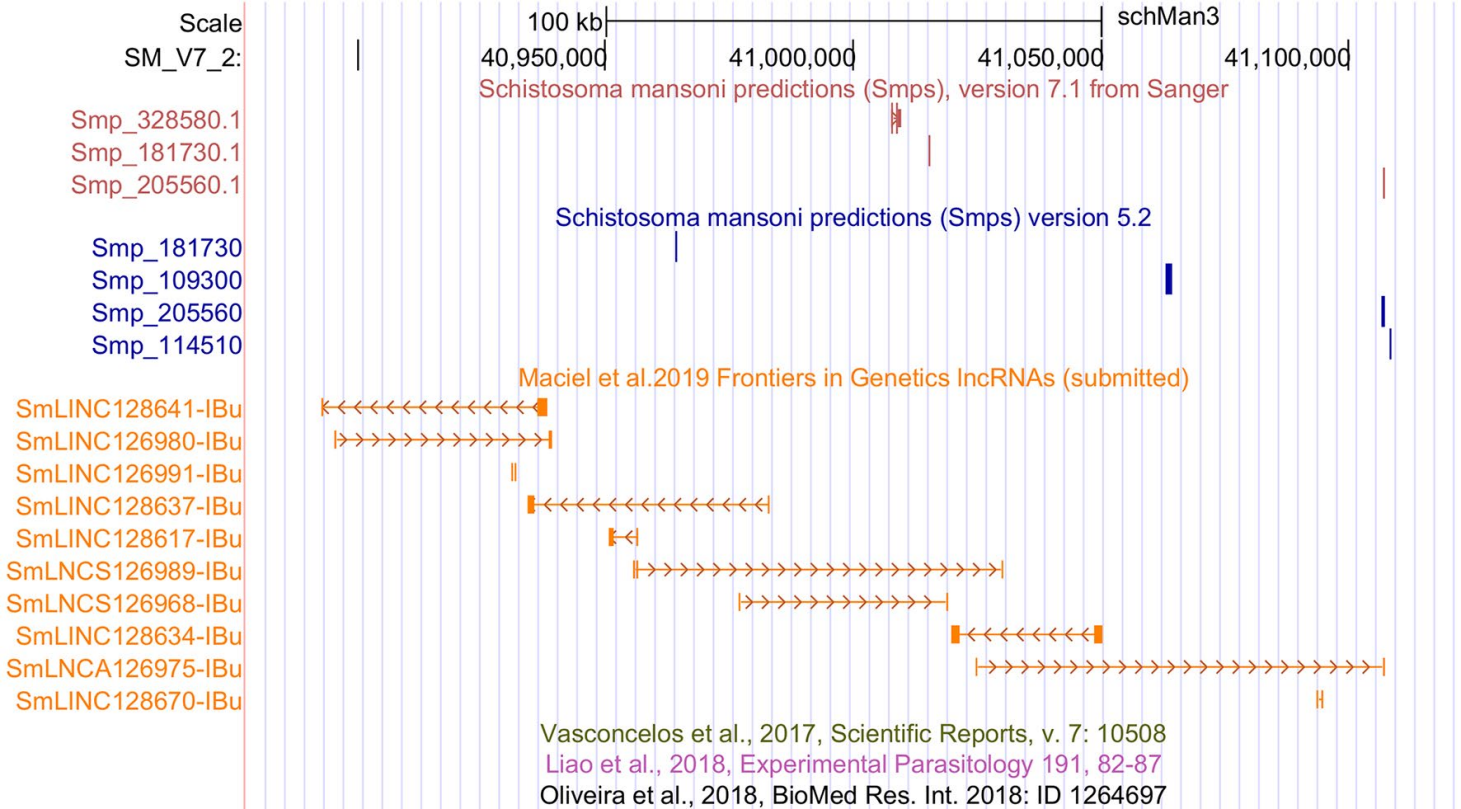
Novel *S. mansoni* lncRNAs discovered in a protein-coding desert *locus*. Snapshot of a *S. mansoni* genome browser image, showing a region spanning 245 kb on chromosome 2 with coordinates SM_V7_2:40,877,676-41,122,371 (top black row). The red track (top) shows the three protein-coding genes from transcriptome version 7.1, whereas the blue track (middle) represents the protein-coding genes from version 5.2. The orange track (lower track) shows seven intergenic lncRNAs (SmLINCnnnnnn-IBu), two sense lncRNAs (SmLNCSnnnnnn-IBu), and one antisense lncRNA (SmLNCAnnnnnn-IBu) that were not annotated by the previously published works on lncRNAs, of which there are three empty tracks at the bottom, namely, Vasconcelos et al. (2017), Liao et al. (2018), and Oliveira et al. (2018).

Among the transcripts in the public data set that were previously classified as lncRNAs (Vasconcelos et al., 2017; Liao et al., 2018; Oliveira et al., 2018) and are now excluded, a total of 4,293 were reconstructed in our assembly; however, they were removed from our set of lncRNAs because they were partially processed pre-mRNA transcripts that have exon-exon overlap with new protein-coding genes of version 7.1. The remaining transcripts previously classified as lncRNAs were reconstructed here but were removed by the more stringent, presently used filtering steps. We have created a track on the *S. mansoni* UCSC-like genome browser (http://schistosoma.usp.br/), where the set of 16,583 lncRNAs obtained here can be visualized and the GTF and BED files can be downloaded. In **Figure 1**, we show a selected protein-coding desert genomic *locus* on chromosome 2 covering 245 kilobases, which harbors only three protein-coding genes and where we identified seven lincRNAs, two sense lncRNAs, and one antisense lncRNA that were not previously described.

**Figure 2:**
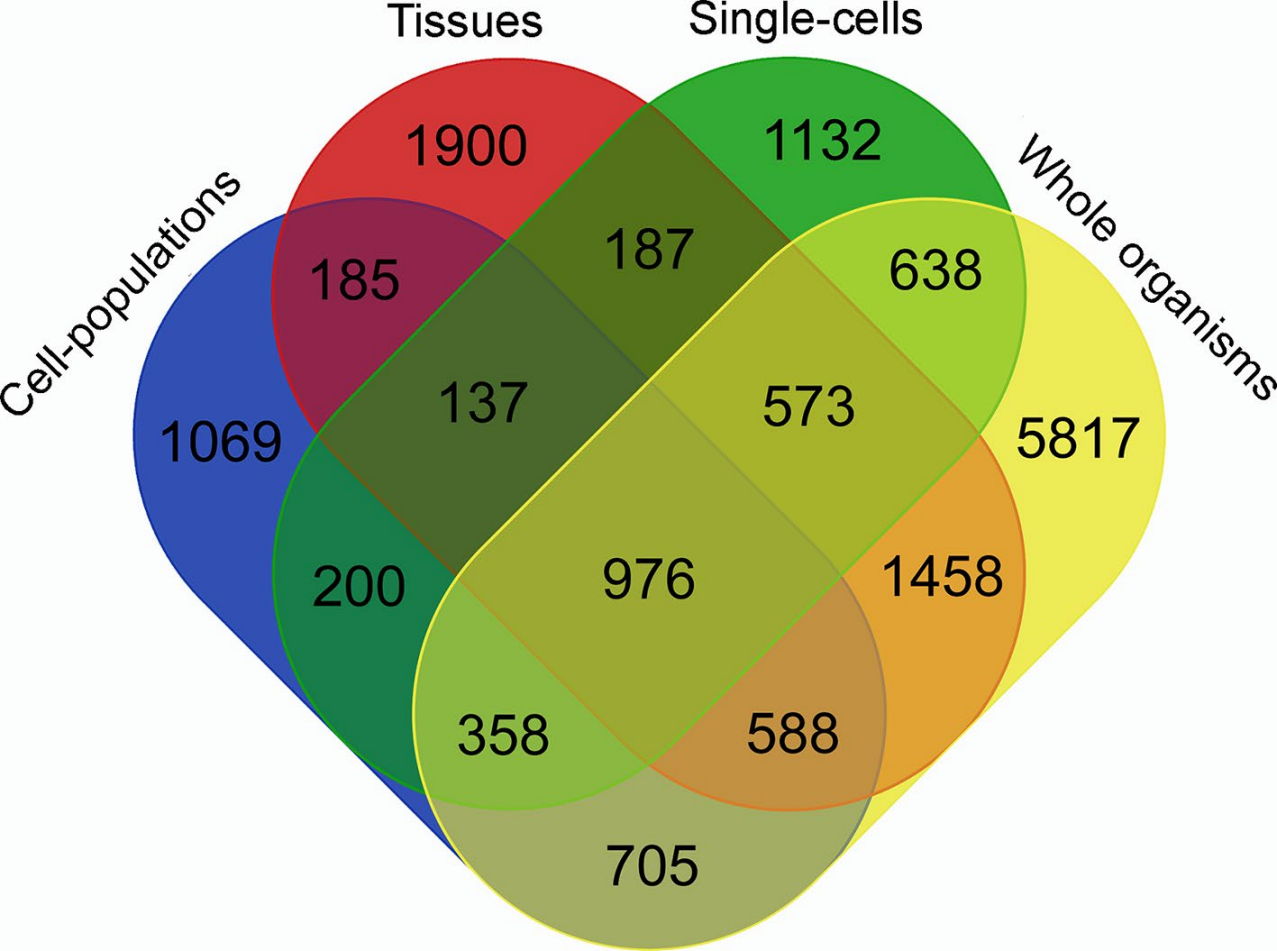
Venn diagram representing the specific contribution from each type of RNA-Seq library to the *S. mansoni* lncRNAs set. TACO assembler was run separately for the RNA-Seq data from samples of four groups: whole organisms (yellow), tissues (red), cell-populations (blue) and single-cells (green), and each value indicates the number of transcripts that were reconstructed specifically with samples from groups indicated in each intersection.

To identify the contribution from each type of RNA-Seq library to the final lncRNAs set, we used the TACO transcriptome assembler to obtain the transcriptomes of the four following groups: whole organisms, tissues, cell populations, and single cells. The result is presented in **Figure 2** and shows that each type of sample contributed with at least 1,000 unique lncRNAs, detected only in that group. It is worthy to mention that around 4% of the 16,583 lncRNAs are lost when the four transcriptomes are reconstructed separately.

**Figure 3:**
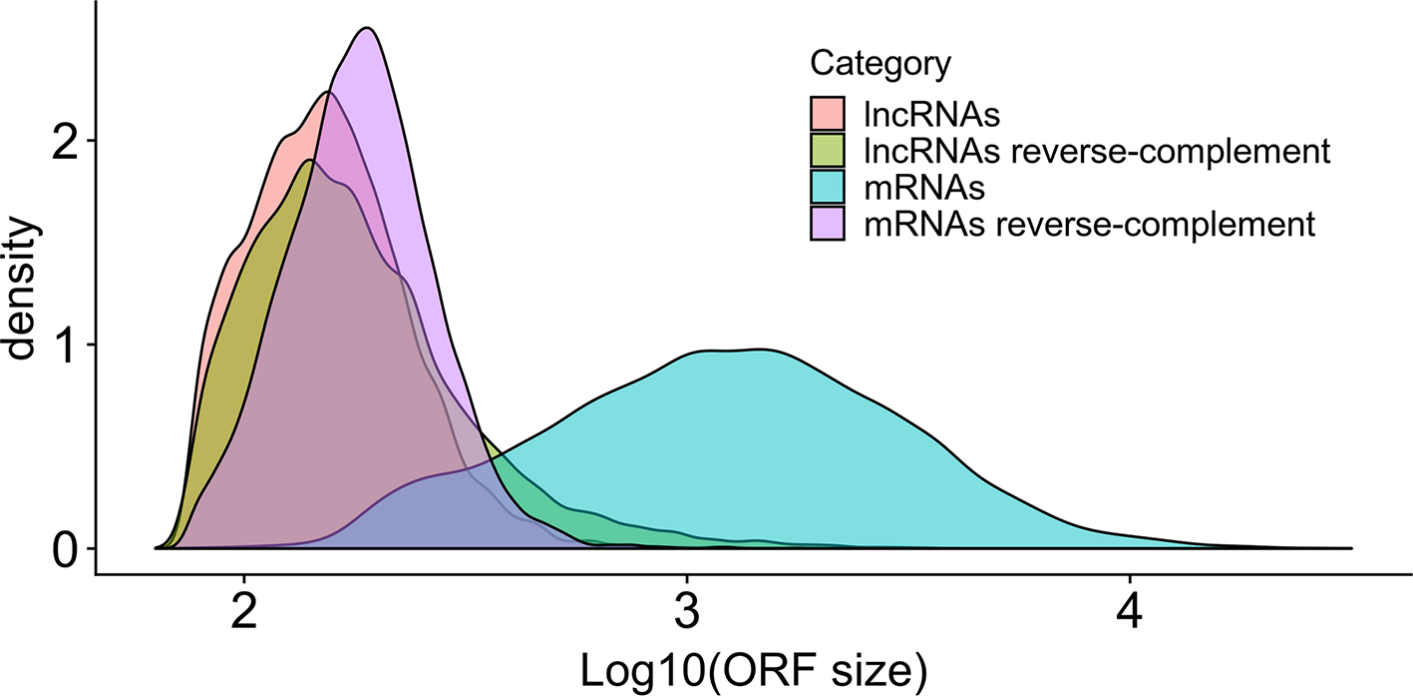
Size distribution in *S. mansoni* of the longest canonical ORFs in lncRNAs and in mRNAs. The graph shows the density (y-axis) of the different sizes for the longest detected ORFs (in nucleotides, x-axis) of all lncRNAs (pink), of all mRNAs (blue) and of their reverse-complement sequences as controls (green and purple, respectively).

Almost all lncRNAs encode short canonical ORFs within their sequences, however, as described by Verheggen et al. (2017), one can evaluate if these ORFs are originated only by random nucleotide progression by comparing the relative sizes of ORFs using the reverse-complement of the sequence as a control. As presented in **Figure 3**, it is very clear that the size distribution of *bona fide S. mansoni* mRNA ORFs (sense) from the annotated v 7.1 transcriptome is greatly shifted toward longer sizes, compared with the size distribution of random ORFs found in their reverse-complement sequences. It is also possible to observe that the size distribution of ORFs found both within the lncRNAs (sense) and within their reverse-complement sequences is very similar and is also similar to the size distribution of random ORFs in the reverse-complement sequence of mRNAs.

### Histone Marks at the TSS of LncRNAs as Evidence of Regulation

**Figure 4:**
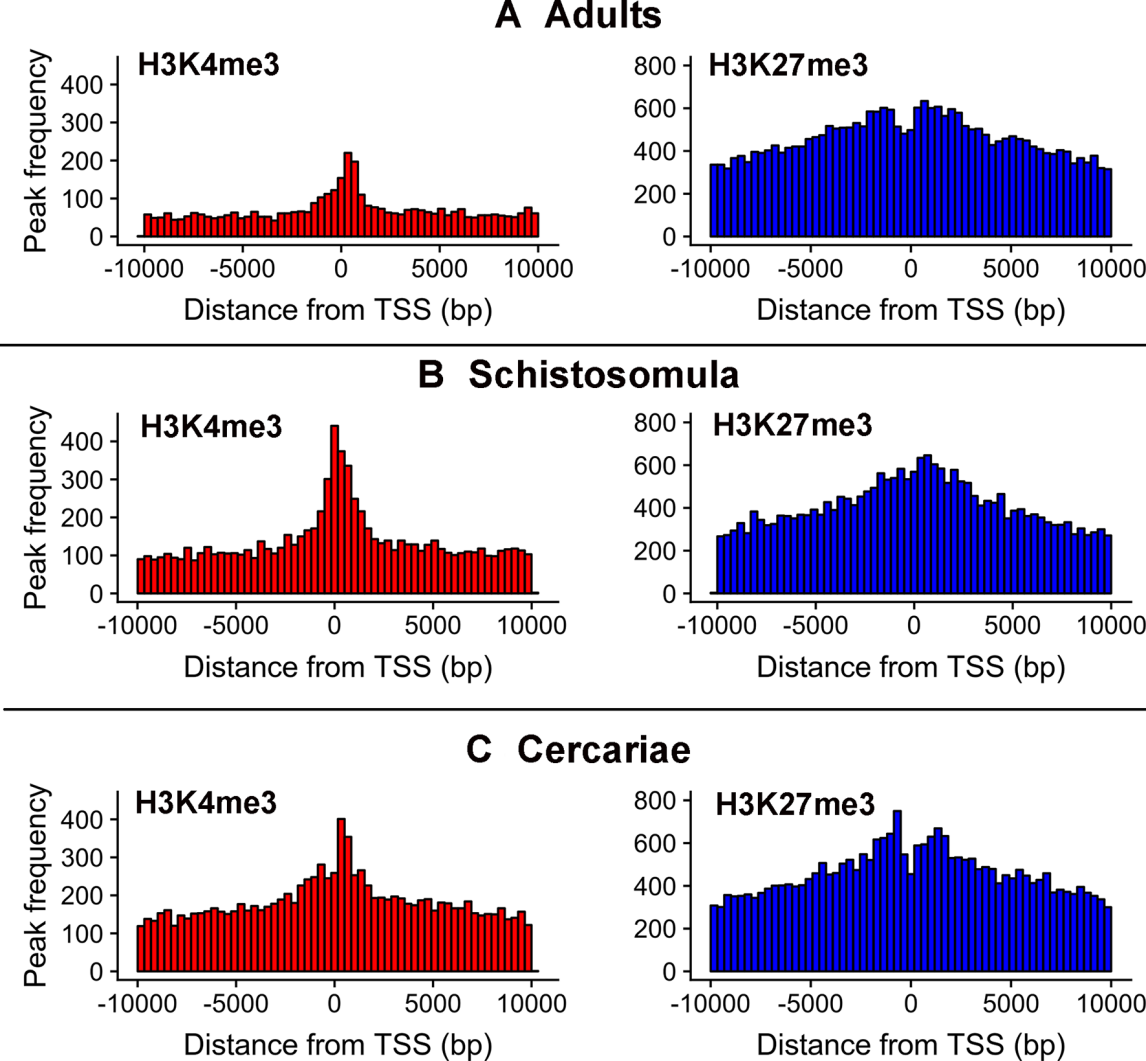
Epigenetic histone marks H3K4me3 and H3K27me3 surrounding the TSS of *S. mansoni* lncRNAs. The frequency of the H3K4me3 marks (red) or of the H3K27me3 marks (blue) mapping within 10 kb around the TSS of all lncRNAs in **(A)** adults, **(B)** schistosomula and **(C)** cercariae was computed.

**Figure 5:**
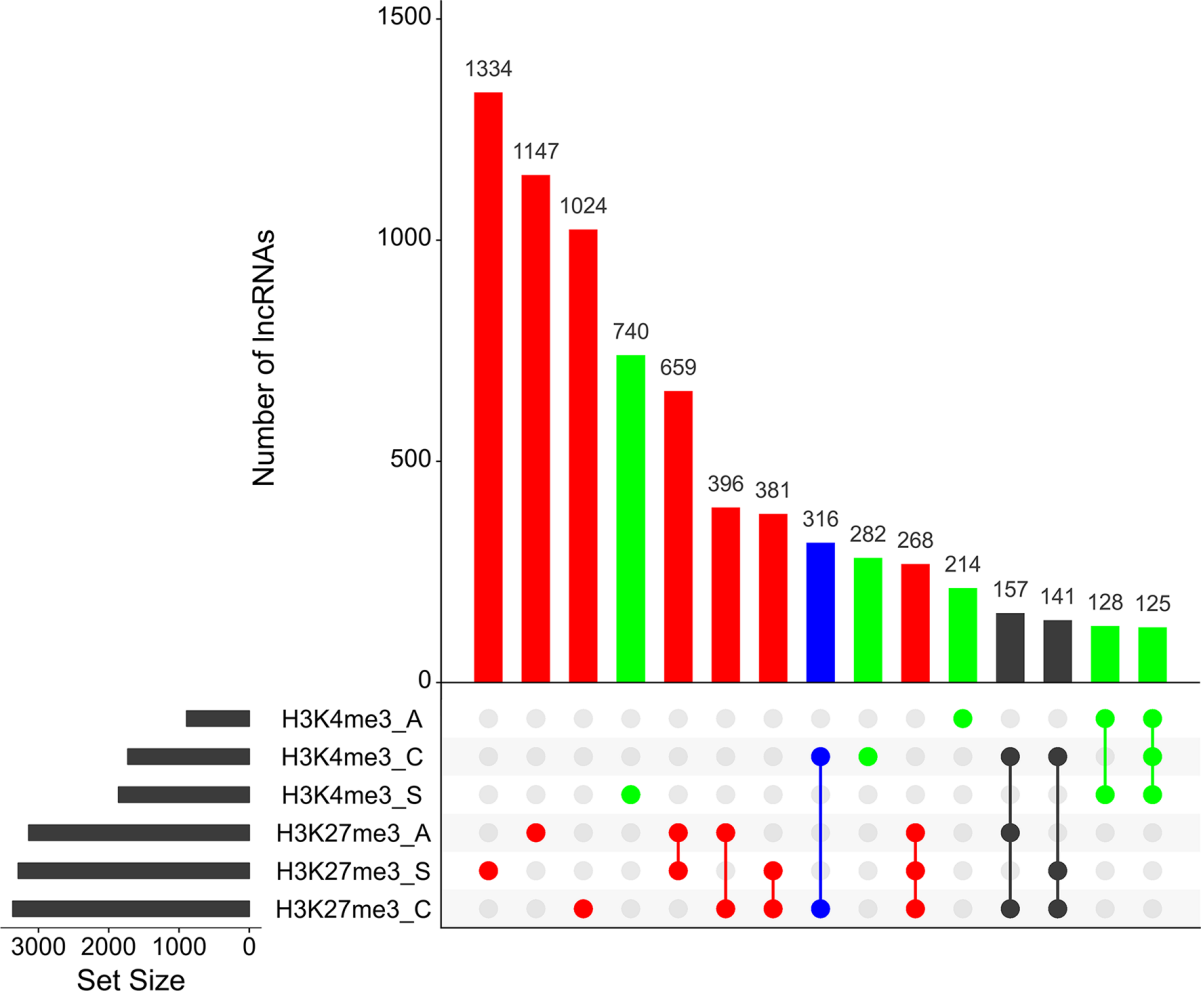
Hundreds of *S. mansoni* lncRNAs have histone transcriptional activating or repressive marks at their TSS exclusively in one life-cycle stage. The UpSet intersection diagram shows the number of *S. mansoni* lncRNAs (y-axis) that have been detected in each of the intersection sets, indicated by the connected points in the lower part of the plot, as having the H3K4me3 transcriptional activating marks (green) and/or the H3K27me3 repressive marks (red) within 1 kb (upstream or downstream) from their TSS. Six histone mark data sets indicated at the bottom left were analyzed: H3K4me3_A in adults, H3K4me3_C in cercariae, H3K4me3_S in schistosomula, H3K27me3_A in adults, H3K27me3_S in schistosomula, and H3K27me3_C in cercariae, and each set size black bar represents the number of lncRNAs that contain the indicated histone mark at the indicated stage. The top 15 most enriched intersection sets are shown; all intersection sets and the lists of lncRNAs in each intersection set are shown in **Supplementary Table S5**. The intersection set in blue shows the number of lncRNAs with the simultaneous H3K4me3_C/H3K27me3_C marks at their TSS in cercariae, characteristic of poised promoters.

As reported earlier, cross-matching of the lncRNAs genomic coordinates with the genomic coordinates of different publicly available histone mark profiles, obtained by ChIP-Seq at different life-cycle stages, adds another layer of functionality evidence for this class of RNAs (Vasconcelos et al., 2017; Cao et al., 2018). We used the data for two different histone marks obtained by Roquis et al. (2018) in cercariae, schistosomula, and adult parasites, namely, H3K4me3 that is generally associated with active transcription, and H3K27me3 associated to transcription repression (Barski et al., 2007). First, we analyzed the histone mark profiles of H3K4me3 and H3K27me3 around the TSS of protein-coding genes through the stages, and they were very similar to the ones presented by Roquis et al. (2018) (**Supplementary Figure S3**). **Figure 4** shows that these marks are also present around the TSS of *S. mansoni* lncRNAs at the three different life-cycle stages; a comparison with **Supplementary Figure S3** shows that these marks are less abundant in lncRNAs than that in the protein-coding genes loci and more spread away of the lncRNAs TSSs when compared with protein-coding genes. This profile is similar to that observed by Sati et al. (2012) when comparing histone marks around the TSS of human protein-coding genes and lncRNAs. A total of 8,599 lncRNA transcripts have at least one histone modification mark within 1 kb from their TSS (**Supplementary Table S5**), being 3,659 lincRNAs, 4,188 antisense lncRNAs, and 752 sense lncRNAs. A comparison of the lists of lncRNAs having a given histone mark at their TSS at either of the three different life-cycle stages (**Figure 5**) shows that the most abundant mark is the transcriptional repressive mark, H3K27me3. This mark is present at the TSS of different sets of lncRNAs at each of the three stages, with abundancies ranging from 1,334 lncRNAs with the H3K27me3 mark exclusively in schistosomula to 1,147 lncRNAs with the mark exclusively in adults and 1,024 lncRNAs with the mark exclusively in cercariae (**Figure 5**, **red**). In addition, the transcriptional activating mark H3K4me3 is present at the TSS of a different set of lncRNAs, with abundancies ranging from 740 lncRNAs with the H3K4me3 mark exclusively in schistosomula to 282 lncRNAs with the mark exclusively in cercariae, and 214 lncRNAs with the mark exclusively in adults (**Figure 5**, **green**). Interestingly, among the lncRNAs with the most abundant patterns of marks at their TSS, there are 316 lncRNAs in cercariae that have the characteristic marks of bivalent poised promoters (having both H3K4me3 and H3K27me3 marks at their TSS) (Voigt et al., 2013) (**Figure 5**, **blue**). This is analogous to the marks at the TSS of protein-coding genes in cercariae, where most genes have the bivalent mark (Roquis et al., 2018), indicating that lncRNAs are under a similar transcriptional regulatory program as the protein-coding genes in cercariae. **Supplementary Table S5** has a complete UpSet plot similar to that of **Figure 5**, showing the number of lncRNAs found in all different intersections, along with the lists of lncRNAs belonging to each intersection set.

### Gene Co-Expression Analyses

Once we identified our final lncRNAs set, we applied weighted gene co-expression network analyses (WGCNA) to integrate the expression level differences observed for lncRNAs and mRNAs among all life-cycle stages and the gonads, using all RNA-seq libraries available. The file containing expression levels (in TPM) for all transcripts in all 633 RNA-Seq libraries is available at http://schistosoma.usp.br/. After normalization and gene filtering (see Methods), 90 libraries out of the 112 from the different stages (mixed-sex adults were not included) remained in the WGCNA analyses, and 19,258 transcripts were retained (12,693 protein-coding genes and 6,565 lncRNAs).

**Figure 6:**
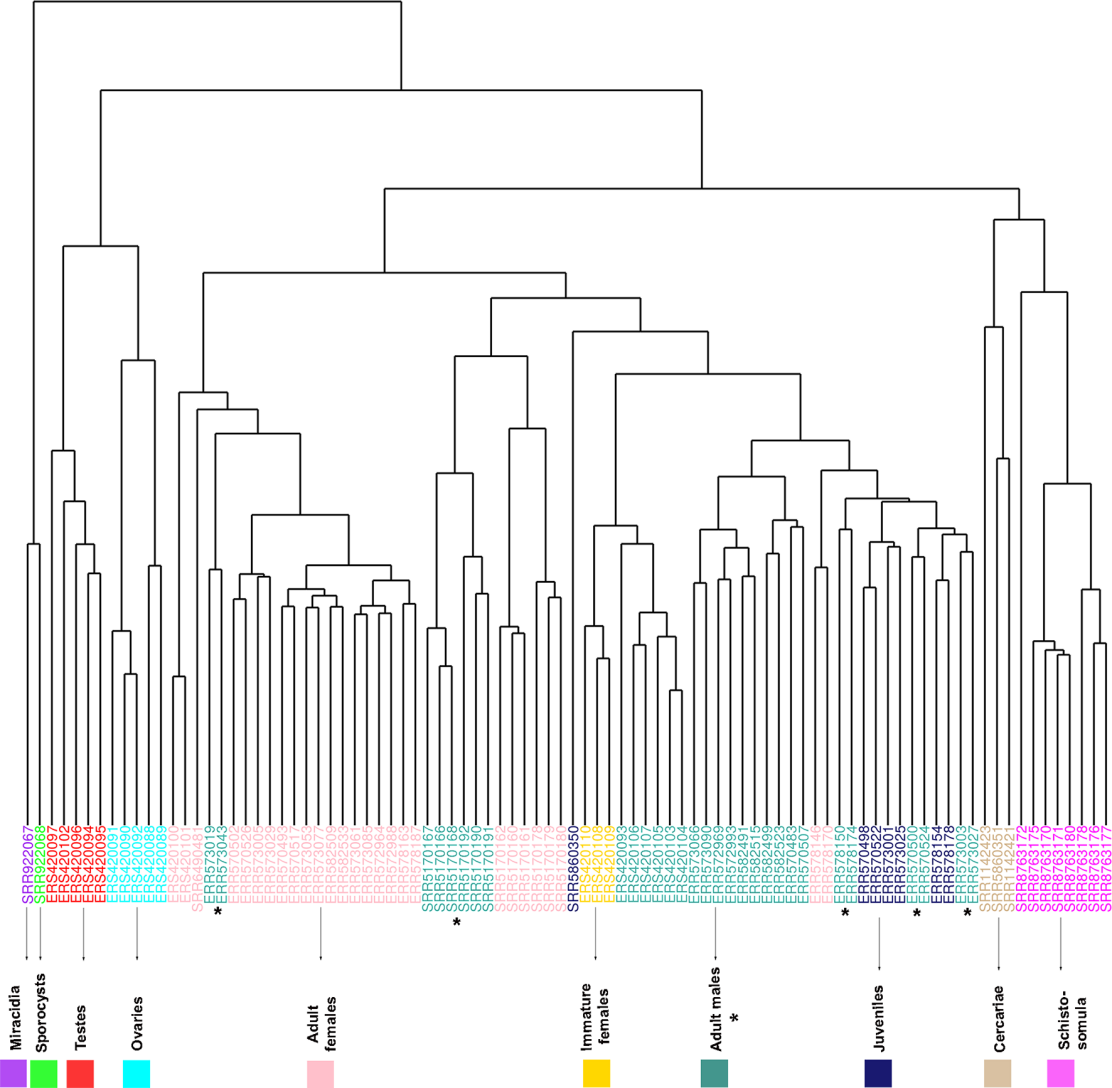
RNA-seq samples clustering based on Euclidian distance according to the expression levels of all genes used for WGCNA. The expression levels of all lncRNA and mRNA genes from all RNA-seq data sets analyzed in the WGCNA were used for unsupervised clustering of the samples, including RNA-seq data sets from adult females (pink), adult males (turquoise), cercariae (tan), immature females (gold), juveniles (midnight blue), miracidia (purple), ovaries (cyan), schistosomula (magenta), sporocysts (green), and testes (red). The SRA or ENA accession number for each RNA-seq library is indicated at each leaf. The asterisks mark the adult male data sets, whose clustering pattern is the most spread one.

Samples from miracidia, sporocysts, schistosomula, cercariae, and gonads (testes and ovaries) were correctly clustered together by their expression correlation, based on Euclidian distance metrics (**Figure 6**). For samples from adult worms, in spite of the fact that we have one cluster branch mainly composed of females, and another mainly composed of males, there are some male samples among the female ones, and vice versa. Besides, due to the known similarity between males and juveniles (Wang et al., 2017), their samples were not well separated. It is interesting to note that immature females, which were shown to have a similar expression profile as that of males (Lu et al., 2016), are clustered here in the male branch. As the WGCNA performs an unsupervised co-expression analysis, we decided to keep all male and female samples in the analysis, including those that are clustered apart from their main group, in order not to add a bias in the construction of modules.

**Figure 7:**
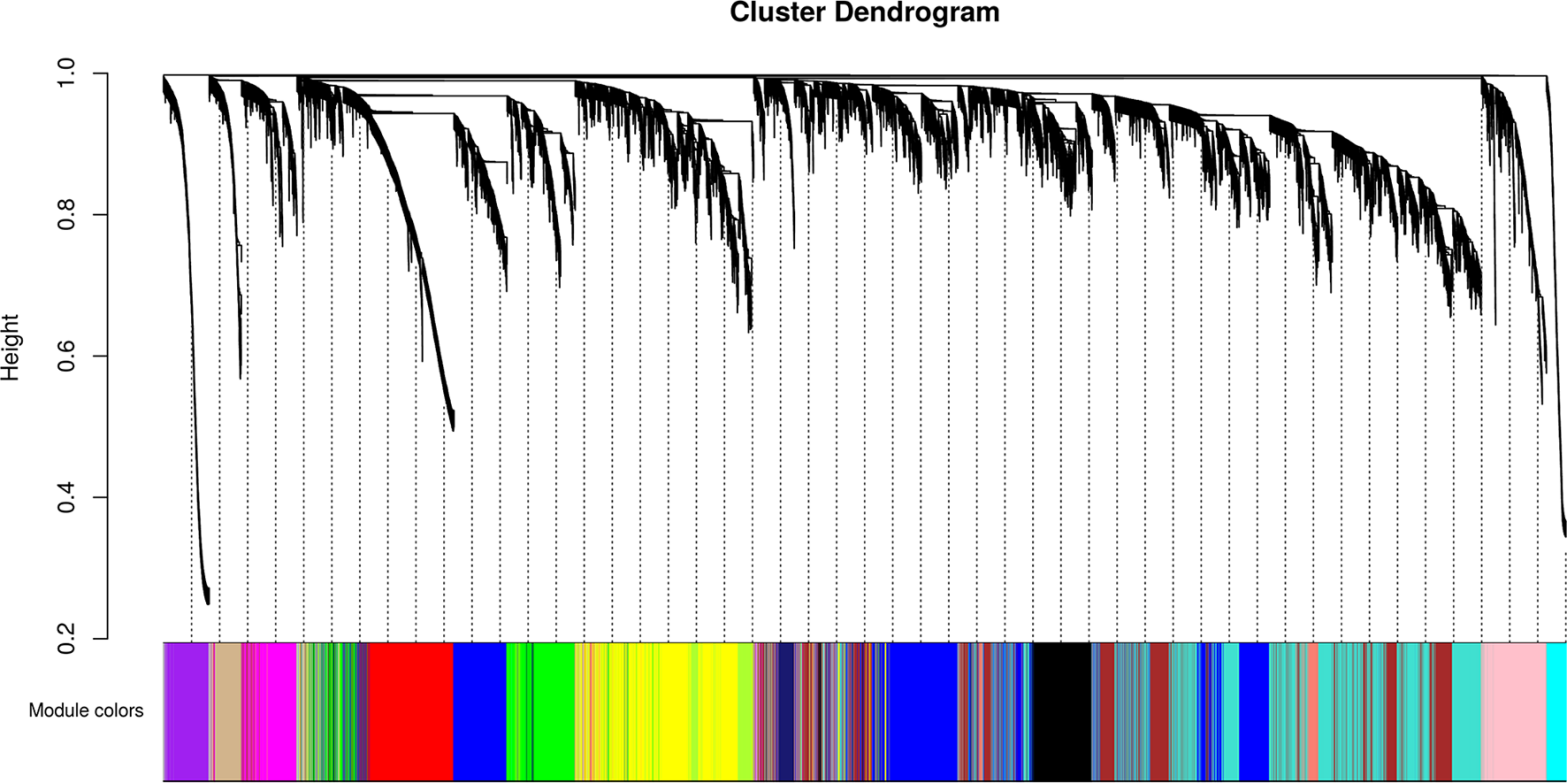
Identification of gene co-expression modules among the different RNA-seq libraries analyzed with the WGCNA tool. Gene hierarchical cluster dendrogram based on a dissimilarity measure of the Topological Overlap Matrix (1 – TOM) calculated by WGCNA, together with the 15 assigned module colors.

We identified 15 different lncRNAs/mRNAs co-expression modules (**Figure 7**), the sizes ranging from 215 to 3,318 transcripts (**Table 2** and **Supplementary Table S6**). The ratio between the number of lncRNAs and mRNAs that comprise each module varies among the modules; thus, whereas lncRNAs comprise 86% of the transcripts in the cyan module, only 5% of the transcripts from the black module are lncRNAs (**Table 2**).

**Table 2:** Number of transcripts per module and percentage of lncRNAs in each module.

Module color	Total number of transcripts	mRNAs	lncRNAs	% of lncRNAs	Stage of higher absolute correlation value
**Black**	989	940	49	5	Miracidia/Sporocysts
**Blue**	3,211	2,688	523	16	Juveniles
**Brown**	2,466	1,761	705	29	Gonads
**Cyan**	253	36	217	86	Ovaries
**Green**	1,308	841	467	35	Gonads
**Greenyellow**	502	417	85	17	Gonads
**Magenta**	748	273	475	64	Schistosomula
**Midnight blue**	215	43	172	80	Juveniles
**Pink**	840	506	334	40	Adult Females
**Purple**	590	274	316	54	Miracidia/Sporocysts
**Red**	1,254	333	921	73	Testes
**Salmon**	267	230	37	14	Gonads
**Tan**	356	158	198	56	Cercariae
**Turquoise**	3,318	2,470	848	26	Adult Males
**Yellow**	2,067	1,398	669	32	Adult Males

**Figure 8:**
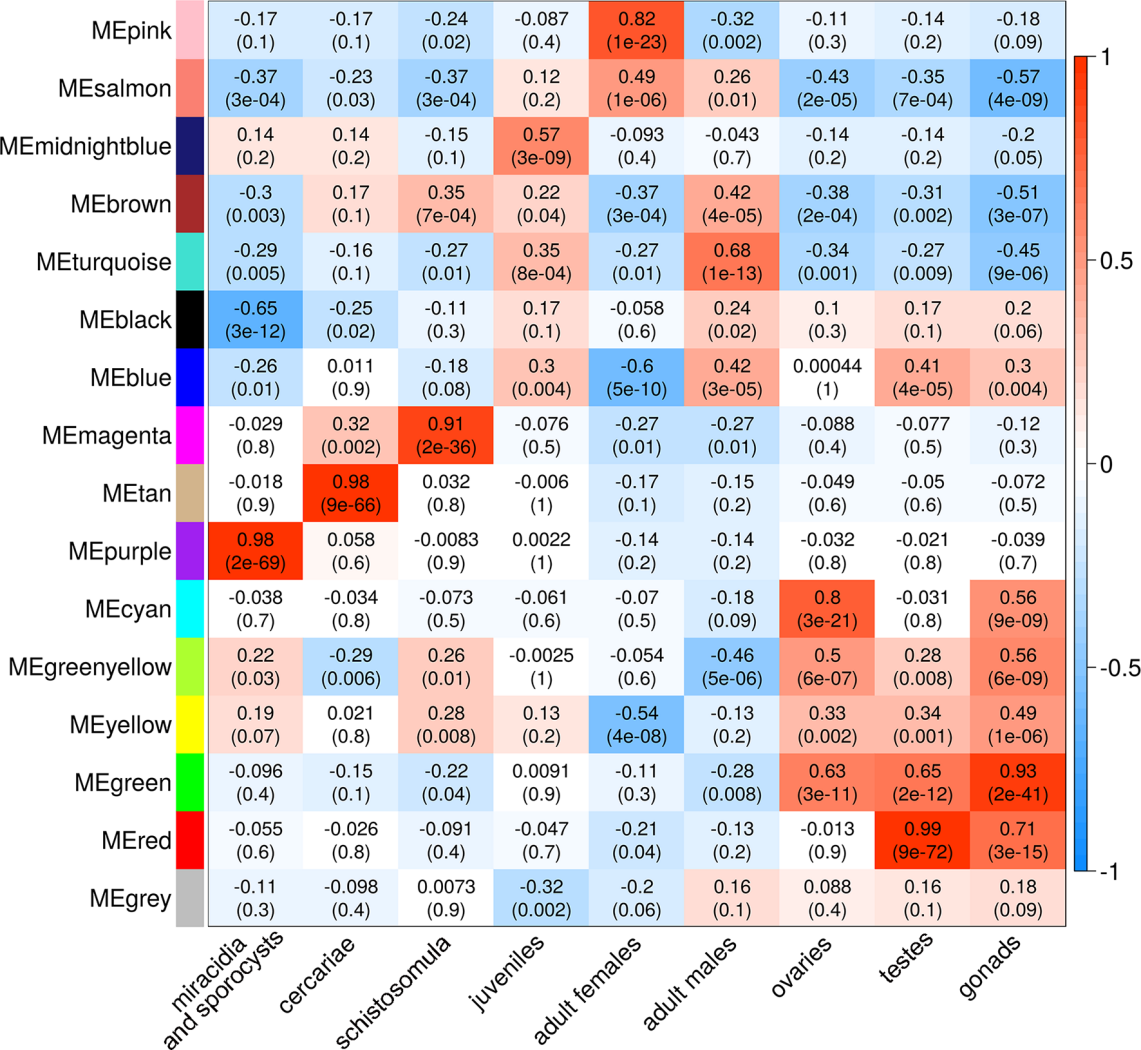
Each parasite life-cycle stage (or tissue) has at least one highly correlated gene co-expression module. Each cell in the table shows the Pearson correlation (with the p-value in parenthesis) between each of the 15 co-expression modules determined by WGCNA (indicated at left) and the stages/tissues of *S. mansoni* (indicated at the bottom). The cells are colored according to the scale (at right), which is related to the Pearson correlation coefficient.

A Pearson correlation analysis indicates that each stage/tissue has at least one module whose gene expression has a statistically significant positive correlation with that stage or tissue (**Figure 8**). Some stages also have modules that have a statistically significant negative correlation, such as the black module that is negatively correlated with miracidia/sporocysts. For the black module, the transcripts that compose the module have an expression in miracidia/sporocysts that is lower when compared with the overall expression of those transcripts across the other stages. The gray color represents the group of transcripts with a highly heterogeneous co-expression pattern that could not cluster into any of the 15 modules. In fact, it can be seen in **Figure 8** that in this group, the best correlation coefficient obtained in juveniles is lower (|r| = 0.32), and the p-value is much higher (p = 0.002) than the best parameters that were obtained in at least one stage for any module (|r| ≥ 0.51 and p ≤ 3e-07). Here, our choice of keeping in the WGCNA analysis, those male and female samples that cluster apart from their main group (**Figure 6**) have an impact, decreasing the correlation coefficient of the modules mostly correlated to males or females (pink or turquoise, respectively) when compared with correlation coefficients in the other stages/tissues, nevertheless, they still have a statistically significant high correlation.

**Figure 9:**
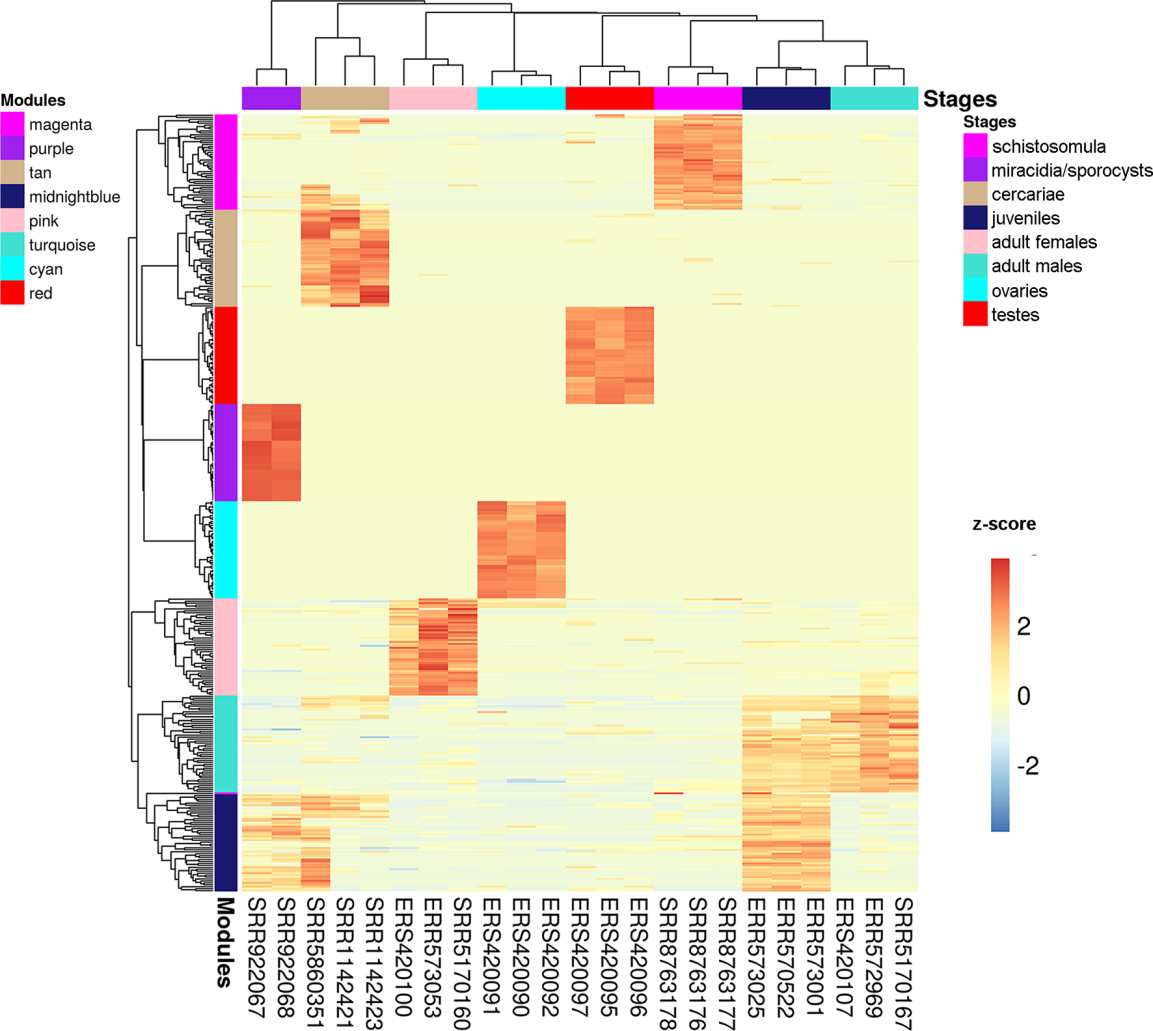
Gene expression heatmap across the life-cycle stages/tissues of the parasite. Representative heatmap of gene expression levels for the top 50 genes (each in one line) with the highest GMM values from each of the eight modules (indicated at the left) with the highest positive correlation to each stage/tissue (indicated at the top). Expression data from three chosen RNA-seq libraries (one in each column) were picked as representative libraries for each stage/tissue, and their SRA or ENA accession numbers are given at the bottom; for miracidia/sporocysts only two RNA-seq libraries were available. Unsupervised clustering using the Euclidean distance was performed; expression of each gene (in one line) is shown as the z-score (from −3 to 3), which is the number of standard deviations above (red) or below (blue) the mean expression value of that gene across all RNA-seq libraries; the z-score color scale is shown on the right.

We chose three RNA-seq library samples from each of the nine different stages/tissues (among all the libraries under analysis) to construct a representative expression heatmap (**Figure 9**). This heatmap shows the expression across all stages of the top 50 transcripts with the highest gene module membership (GMM) to the most correlated module of each stage (as seen in **Figure 8**) (for GMM definition see WGCNA background and glossary, available at https://horvath.genetics.ucla.edu/html/CoexpressionNetwork/Rpackages/WGCNA/Tutorials/) (Langfelder and Horvath, 2008). The heatmap (**Figure 9**) confirms that the top transcripts belonging to one module are more expressed in one given stage/tissue, which is the stage/tissue with which the module has the highest correlation. It is noteworthy that female library SRR5170160, which clustered inside the male group (**Figure 6**) when all filtered transcripts under analysis were used for clustering, now is correctly clustered with the other female samples (**Figure 9**) when only the top 50 transcripts with the highest GMM are considered. Also, juveniles share with adult males a similar expression pattern of the top 50 male genes, which is in line with the clustering of juveniles along with males in the analysis of **Figure 6**.

### Validation of lncRNAs Expression by RT-qPCR

We designed PCR primer pairs for a selected set of eleven lincRNAs belonging to five different modules, as determined by WGCNA, to detect their expression along the different *S. mansoni* life-cycle stages and to eventually validate their different expression levels at the stages. Our selection was based on the Gene Trait Significance score (GS score) (**Supplementary Table S7**) of each lncRNA in the module where it belongs, which varies from −1 to 1, using the stages as external information (see Methods). The higher the absolute value of the GS score, the more biologically significant and correlated to the stage of interest is the transcript expression. For the RT-qPCR assays, we used samples from eggs (E), miracidia (Mi), cercariae (C), schistosomula (S), adult males (M), and females (F).

First, we measured the expression of five protein-coding genes that were used as stage markers (Parker-Manuel et al., 2011; Anderson et al., 2016), and we found that in our RNA samples, they were more highly expressed at the predicted stages (**Supplementary Figure S4**).

**Figure 10:**
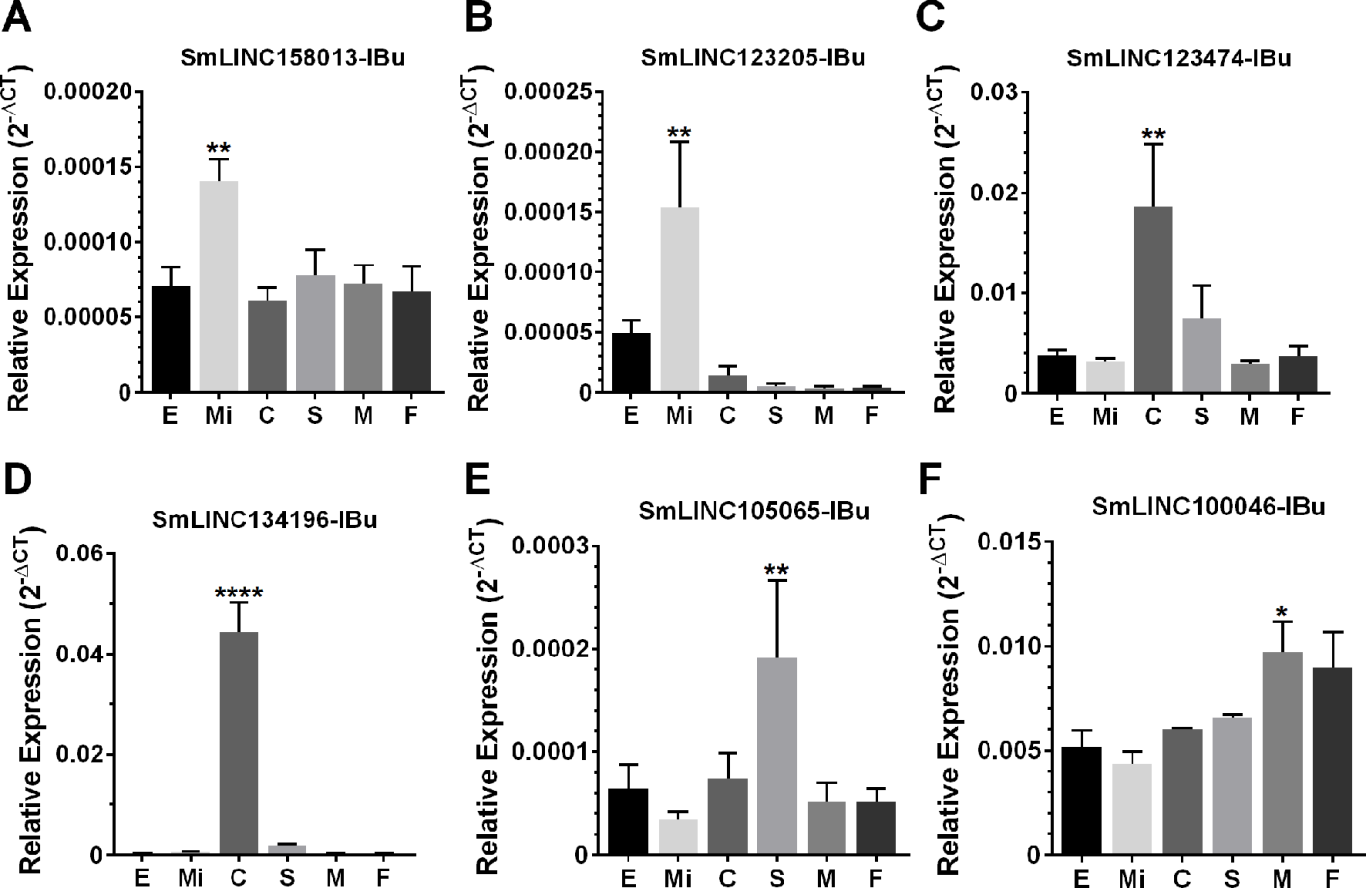
Confirmation by RT-qPCR of the module-specific lincRNAs relative expression. Six lincRNAs were measured at different developmental stages of *S. mansoni*. From left to right in the x-axis, lincRNAs were measured in RNA samples from eggs (E), miracidia (Mi), cercariae (C), *in vitro* mechanically transformed schistosomula cultivated for 24 h (S), adult males (M) and females (F). The lincRNAs relative gene expression was calculated against the geometric mean of two housekeeping genes: Smp_090920 and Smp_062630. **(A)** and **(B)** show SmLINC158013-IBu and SmLINC123205-IBu representing the **purple** module, specific for miracidia/sporocysts. In **(C)** and **(D)**, the SmLINC123474-IBu and SmLINC134196-IBu representing the cercariae-specific **tan** module. In **(E)**, the schistosomula-specific lincRNA SmLINC105065-IBu from the **magenta** module and **(F)** the adult male-specific lincRNA SmLINC100046-IBu from the **turquoise** module. Bars represent standard deviation of the mean from four biological replicates for each stage. Two technical replicates were assayed for each of the four biological replicates per stage. The ANOVA Tukey test was used to calculate the statistical significance of the expression differences among the parasite stage samples (*p value ≤ 0.05; **p value ≤ 0.01; ****p value ≤ 0.0001). For clarity purposes, we show only the highest p value obtained in the ANOVA Tukey test for expression comparisons against one another among the stages.

Then, we tested the selected eleven lincRNAs and detected that they were expressed in at least one of the six stages that were assayed; specifically, each of six lincRNAs were more highly expressed at the stage predicted by the correlation with the modules (**Figure 10**), at four life-cycle stages: two more highly expressed in miracidia (SmLINC158013-IBu and SmLINC123205-IBu, purple module), two in cercariae (SmLINC123474-IBu and SmLINC134196-IBu, tan module), one in schistosomula (SmLINC105065-IBu, magenta module), and one in males (SmLINC100046-IBu, turquoise module) (**Figure 10**). In **Supplementary Figure S5** we present the values in transcripts per million reads (TPM) from the RNA-seq libraries for each of these six validated lincRNAs. Additionally, the five other lincRNAs that were tested were detected as expressed across all stages; however, they were not differentially expressed as predicted by the RNA-seq (**Supplementary Figure S6**). This indicates that there is variability of lncRNAs expression between the experimental conditions and parasite strain used in our assays and those found among the dozens of samples that are publicly available.

### Protein-Coding Genes Ontology Enrichment and lncRNA Hub Genes in the Modules

**Figure 11:**
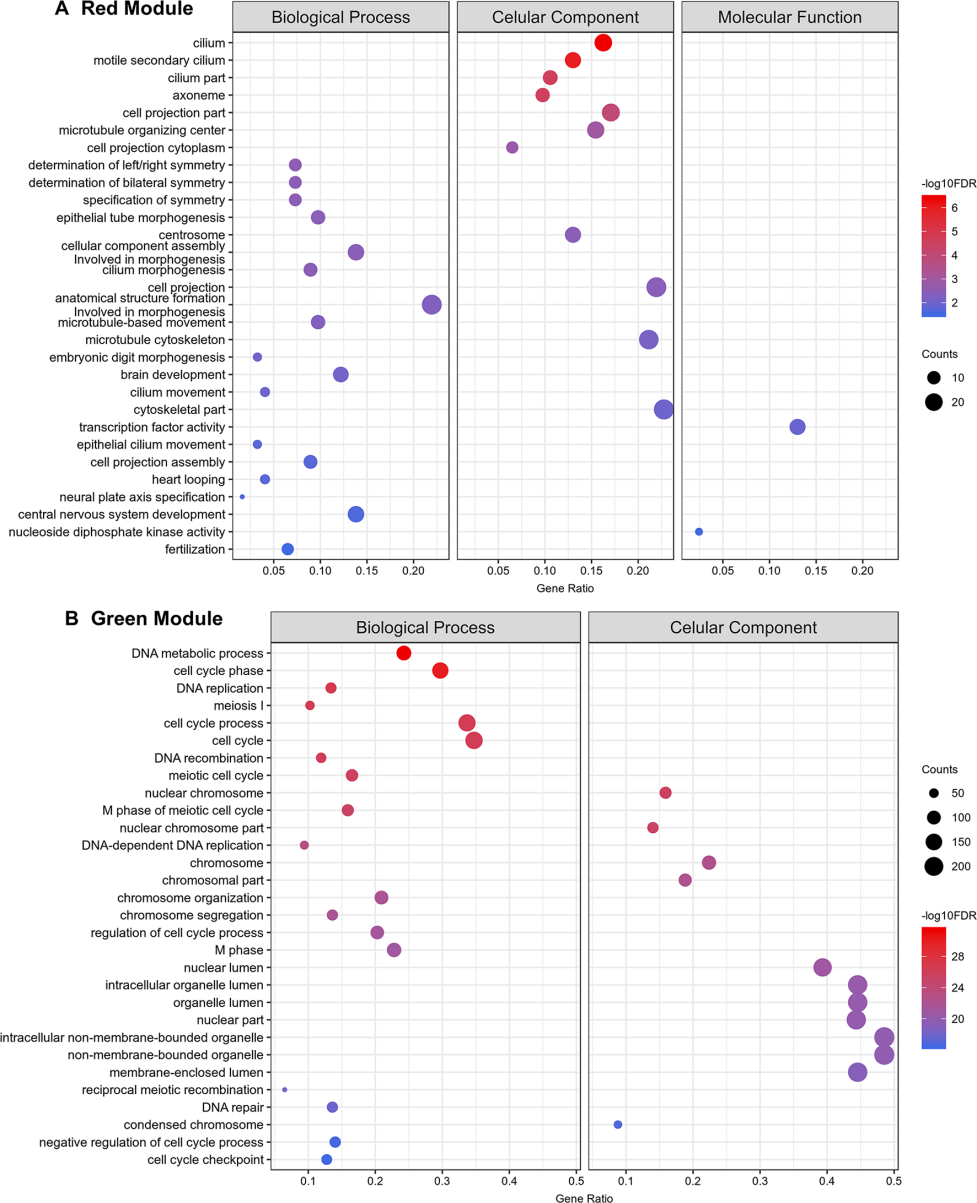
Top 30 Gene Ontology most significantly enriched terms for protein-coding genes belonging to the red and green co-expression network modules. At left are the enriched GO term annotations. For the **(A)** red (testes) and the **(B)** green (gonads) modules, the enriched GOs are separately represented into the three major GO term categories, namely Biological Process, Cellular Component and Molecular Function. No Molecular Function term was significantly enriched in the green module. The size of the circles is proportional to the number of genes (counts scale on the right) in each significantly enriched GO category, and the colors show the statistical significance of the enrichment, as indicated by the -log10 FDR values (color-coded scales at the right).

Gene ontology (GO) enrichment analyses show that the protein-coding genes belonging to the red module, which have a correlation of 0.99 with testes, are enriched with processes related to sperm motility such as cilium movement and the axoneme assembly (**Figure 11A**). Besides, the green module, correlated with both ovaries and testes, is enriched with proteins associated with cellular replication (**Figure 11B**). All other modules with GO enrichment, which in general are enriched with proteins associated to general metabolism, are presented in **Supplementary Figures S7–S10**. The black, cyan, midnight blue, purple, and tan modules have no significantly enriched GO terms due to the small number of protein-coding genes with GO annotation within each of these modules.

**Figure 12:**
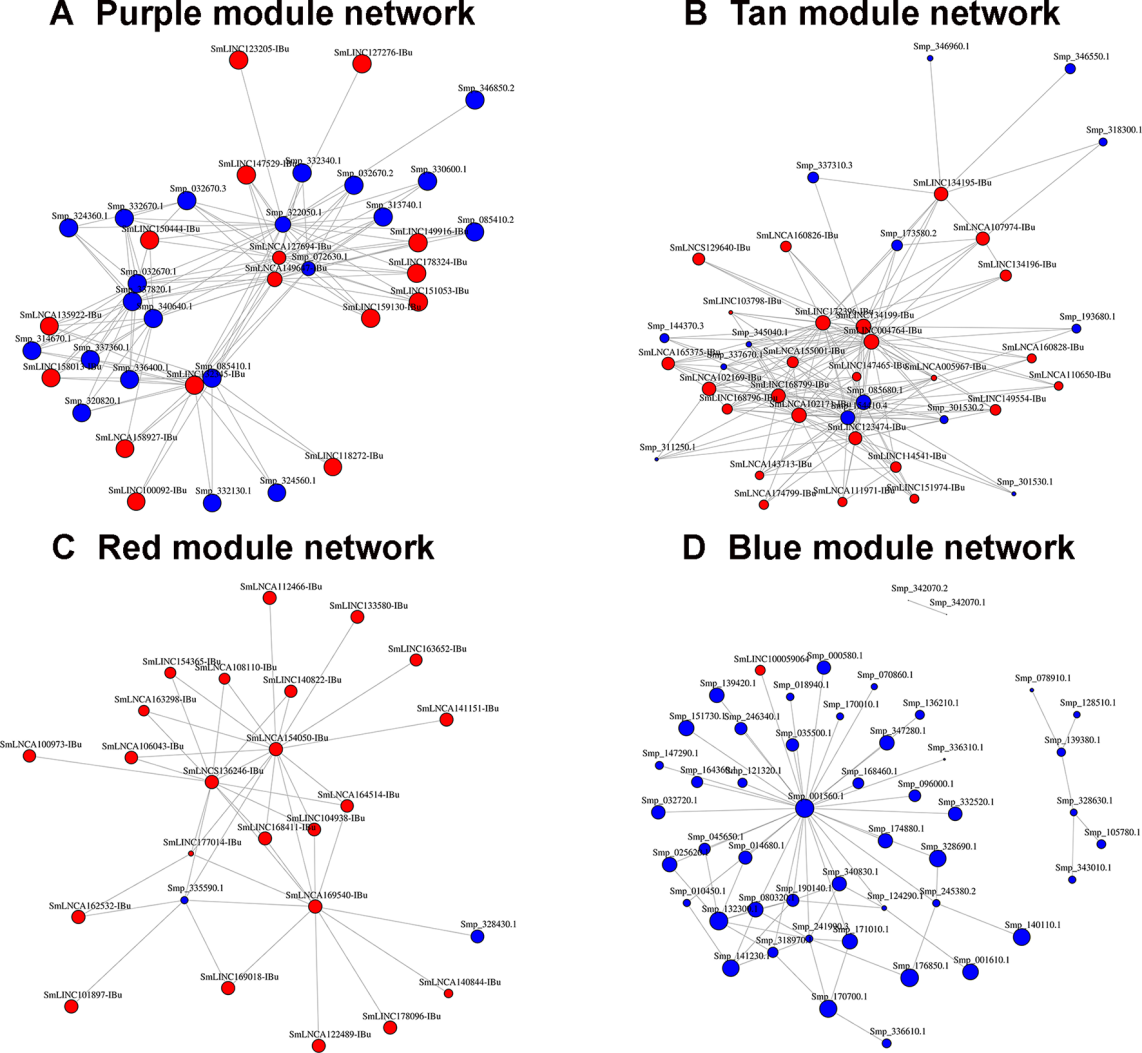
Gene co-expression networks. The top most highly connected genes from the **(A)** purple (miracidia/sporocysts), **(B)** tan (cercariae), **(C)** red (testes) or **(D)** blue (females) co-expression network modules are shown. The adjacency thresholds are 0.77, 0.48, 0.58, and 0.26, respectively. Each red circle represents one lncRNA (SmLINC, SmLNCA, or SmLNCS), and each blue circle represents one protein-coding gene (Smp_). Circle sizes are related to the intramodular connectivity value for each transcript.

All transcripts that belong to the same module are connected; however, to better visualize this, gene co-expression networks were constructed only with the most connected genes (as determined by the adjacency threshold) (**Figure 12**), and they show, along with the correlation values presented in **Supplementary Table S7**, that some lncRNAs are hub genes from the network. **Figures 12A, B** show lncRNA hub genes in the co-expression networks from the purple and tan modules strongly correlated with miracidia/sporocysts and cercariae life-cycle stages, respectively. In both modules, the lncRNAs represent around half of the transcripts that comprise the modules (see **Table 2**). However, there are some cases, such as in the red module, where three quarters of the member transcripts are lncRNAs, and among the most connected genes in that co-expression network, almost all are lncRNAs (**Figure 12C**). Also, in the blue module only, 16% of the member transcripts are lncRNAs, and only one is among the most connected genes in the co-expression network (**Figure 12D**). All the gene networks for all modules in a format compatible with Cytoscape are available at **Supplementary Table S8**. An adjacency cutoff threshold of 0.1 was used.

### LncRNAs Expressed in Single Cells

**Figure 13:**
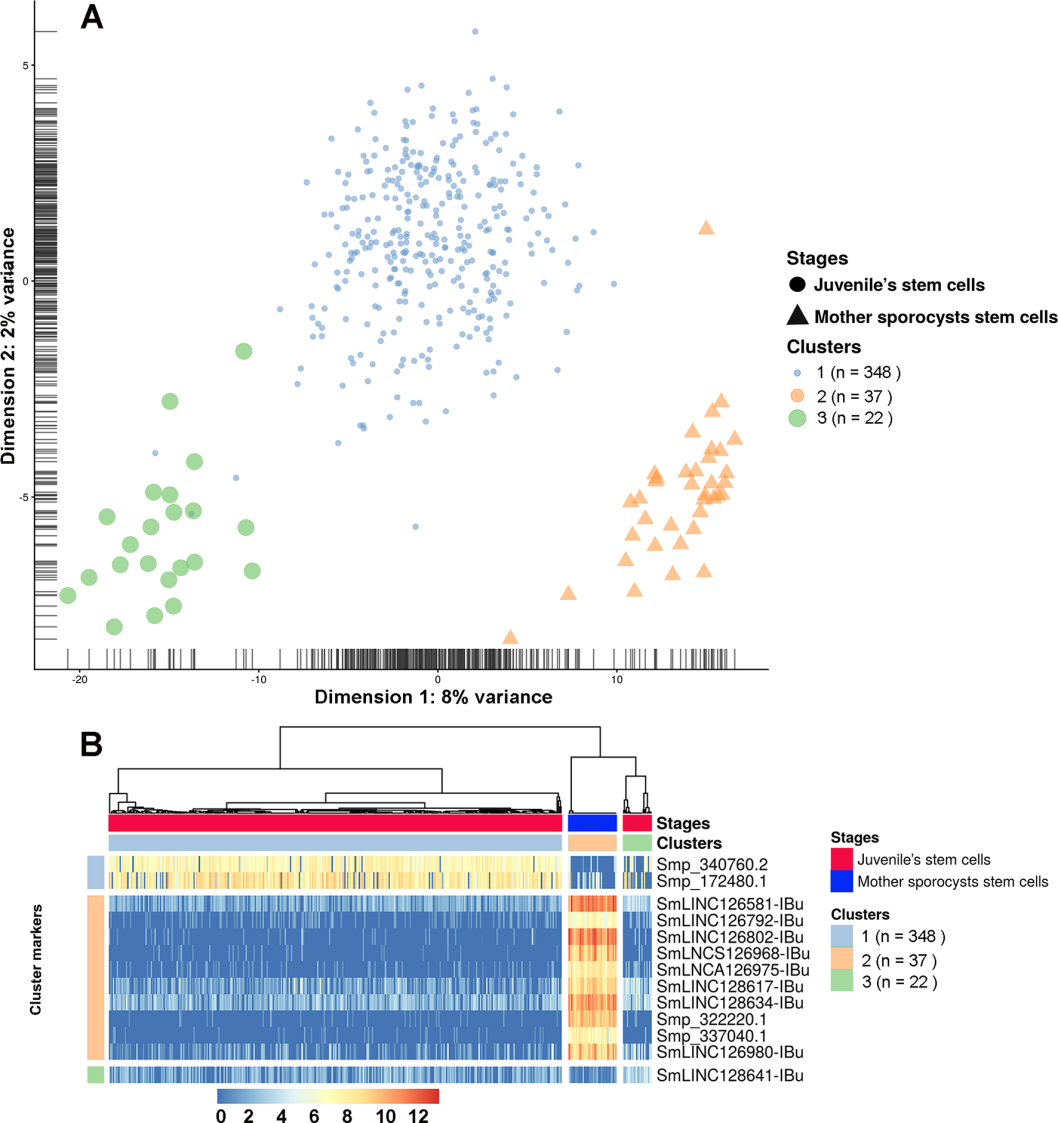
Single-cell expression analysis identified three different cell population clusters when comparing *S. mansoni* juveniles’ stem cells and mother sporocysts stem cells and lncRNAs as gene markers at the single-cell level. **(A)** Single-cell RNA-Seq data from two RNA-Seq libraries, one from juveniles’ stem cells and another from mother sporocysts’ stem cells, were analyzed with the SC3 tool that performed an unsupervised clustering of the cells based on the single-cell gene expression data. Principal component analysis plot, where the symbol colors and sizes indicate the three clusters identified by SC3, and the shapes indicate the two life-cycle stages from which the stem cells were isolated. The symbol size is inversely related to the number of cells that belong to the cluster. **(B)** In the marker-gene expression matrix (log-transformation, represented by the color scale), the statistically significant gene markers are the rows, and the cells are columns. The life-cycle stage from which each cell was isolated is indicated by the color bar at the top (stages). The clusters of cells are separated by white vertical lines and are indicated by the second color bar at the top (clusters). The cluster marker genes are separated by white horizontal lines, the markers groups are indicated at left, and the names of the marker genes at right. Only the top 10 most significant marker genes are shown for cluster 2.

Finally, analyses using single-cell data from two stages, mother sporocysts stem cells and juveniles’ stem cells, identified three different clusters. Cluster 1 is composed of a subgroup of juvenile stem cells, cluster 2 is composed of all mother sporocysts stem cells, and cluster 3 is composed of a second and smaller subgroup of juvenile stem cells (**Figure 13A**). The marker gene analyses show, for the first time in *S. mansoni*, that lncRNAs have specific expression also at the single-cell level, where from the top 10 markers that allow us to differentiate mother sporocysts stem cells from juvenile stem cells, eight are lncRNAs (**Figure 13B**), confirming the stage specificity of lncRNAs also seen in whole worm analyses by WGCNA. Besides, another lncRNA was identified as a marker for cluster 3 when compared with the other two clusters (**Figure 13B**).

## Discussion

When the human genome was first sequenced, the vast genomic regions that lie between protein-coding genes (intergenic regions) were considered junk DNA; one decade later, the Encyclopedia of DNA Elements (ENCODE) project found that 80% of the human genome serves some biochemical purpose (Pennisi, 2012), including giving rise to the transcription of nearly 10,000 lncRNAs (Derrien et al., 2012). Although we are still at the beginning of the studies with lncRNAs, with the vast majority of their roles and mechanisms of action in human beings still unknown, it is now clear that most of the lncRNAs are transcribed from intergenic regions and are key regulators in vital processes (Kitagawa et al., 2013; Rosa and Ballarino, 2016; Golicz et al., 2018), being associated to several pathologies in humans, such as cancer (Fang and Fullwood, 2016), Alzheimer’s (Zijian, 2016), and cardiac diseases (Simona et al., 2018).

In *S. mansoni*, with the release in 2012 of version 5.2 of the genome and annotated transcriptome (Protasio et al., 2012), and with the accumulation until 2017 of large amounts of information on gene expression obtained through 88 publicly available RNA-seq libraries, our group decided to map the RNA-seq data and identify the lncRNAs repertoire expressed in this parasite (Vasconcelos et al., 2017); this was followed by two other papers that provided an additional set of lncRNAs (Liao et al., 2018; Oliveira et al., 2018). In the present work, by extending the analysis to 633 publicly available RNA-seq libraries, and by performing a detailed curation of the assembled transcripts, we observed that at the sequencing depth obtained with the current RNA-seq data sets, a considerable amount of partially processed pre-mRNAs is being sequenced. These pre-mRNAs give rise to assembled transcript units showing intron retention and frequent stop codons in the retained introns, and therefore, these transcripts can be mistakenly annotated as lncRNAs.

In fact, the failure to identify partially processed pre-mRNA in previous publications (Vasconcelos et al., 2017; Liao et al., 2018) may explain the report of probable protein-coding genes as lncRNAs (**Supplementary Figure S1**). Our current pipeline has removed at step 4 a total of 31,183 assembled transcripts that had partial or total exon-exon overlap on the same genomic strand with known *S. mansoni* protein-coding genes, and this included around 14,000 assembled transcripts that represented fully processed mature protein-coding transcripts that exactly matched the annotated v 7.1 transcripts from the Wellcome Sanger Institute, as well as some 17,000 assembled transcripts that for the most part represent partially processed pre-mRNAs with intron retention; among the latter are 4,293 transcripts that were previously classified as lncRNAs (Vasconcelos et al., 2017; Liao et al., 2018; Oliveira et al., 2018) and are now excluded. With the six stringent filtering steps used in the present work, we are confident that our final set of 16,583 lncRNAs is a robust representation of the lncRNAs complement expressed in *S. mansoni*, of which 11,022 transcripts are novel lncRNAs, and 5,561 have gene overlap with lncRNAs already reported in previous works (Vasconcelos et al., 2017; Liao et al., 2018; Oliveira et al., 2018).

One question that has been raised about lncRNAs is the possibility that their function is executed through translation into short peptides, a concern that arises from the fact that almost all lncRNAs encode short canonical ORFs within their sequences (Verheggen et al., 2017); the fact that the size distribution of ORFs found within our set of lncRNAs (sense) is very similar to the size distribution of random ORFs found within their reverse-complement sequences and within the reverse-complement sequence of mRNAs suggests that the putative short ORFs from the lncRNAs identified here are indeed random ORFs, most probably not translated into short functional peptides. Nevertheless, future functional characterization in *S. mansoni* of selected lncRNAs may eventually include a search for a possible dual function role (Nam et al., 2016; Choi et al., 2018) both as lncRNA and through a translated short peptide.

Histone marks were found here at the TSS of lncRNAs, and the identification of different sets of lncRNAs that have at their TSS the transcriptional activation H3K4me3 mark, or the repressive H3K27me3 mark, when the three life-cycle stages are compared, suggests that lncRNAs expression in *S. mansoni* is regulated by an epigenetic program. This finding reinforces the hypothesis that different lncRNAs may play important roles along the parasite life-cycle, and the sets of lncRNAs identified in this analysis might be the first candidates to be explored for further functional characterization.

Gene co-expression networks correlated to the different *S. mansoni* life-cycle stages were identified by our analyses, and they pointed to sets of protein-coding genes and lncRNAs with expression most correlated to one given stage. This information provides an initial platform for prioritizing the lncRNAs to be selected for further direct functional characterization, which will include a search for altered *S. mansoni* phenotypes upon knockdown of lncRNA candidates. In *Plasmodium falciparum*, the knockdown of antisense lncRNAs has down-regulated the active var gene, a gene related to immune evasion, erasing the epigenetic memory and substantially changing the var gene expression pattern (Amit-Avraham et al., 2015). In analogy, it is expected that characterization of lncRNAs in *S. mansoni* will help to recognize the biochemical pathways where they play a functional role, will permit to identify their interacting protein partners, and will eventually point to relevant ways of intervention in the parasite physiology.

Due to the complex and diverse mechanisms displayed by lncRNAs in regulating protein-coding genes and miRNAs, the majority of studies have not progressed beyond cell or animal models, and progression toward the clinic has been slow (Harries, 2019). Nevertheless, lncRNAs represent potentially good therapeutic targets (Matsui and Corey, 2017; Blokhin et al., 2018; Harries, 2019). As reviewed by Matsui and Corey (2017), in Angelman syndrome model mouse, the administration of antisense oligonucleotides (ASOs), which target the Ube3a‐ATS lncRNA for degradation, partially reversed some cognitive defects associated with the disease in the animals (Meng et al., 2014). Also, in xenograft melanoma models, the intravenous injection of ASOs targeting the lncRNA SAMMSON caused p53 activation, tumor growth suppression, decreased cell proliferation, and increased apoptosis (Leucci et al., 2016). In this respect, it is noteworthy that lncRNAs are considerably less conserved between species when compared with protein-coding genes (Pang et al., 2006; Blokhin et al., 2018), and that only a few dozen ancient lncRNAs have conserved orthologs between ancient non-amniote *Xenopus* and the closest amniote chicken model animals (Necsulea et al., 2014), which shows that lncRNAs have evolutionarily conserved gene regulatory functions but low-sequence conservation across distant species (Necsulea et al., 2014). This feature reduces the chances that targeting a lncRNA in *S. mansoni*, for example, with ASOs, will cause unwanted off-target effects against the mammalian host.

## Data Availability

The data sets analyzed in this study can be found in the SRA repository (https://www.ncbi.nlm.nih.gov/sra) and in the ENA repository (https://www.ebi.ac.uk/ena). The specific accession numbers for each and all data sets that were downloaded from these databases and used here are given in **Supplementary Table S1**.

## Ethics Statement

All protocols involving animals were conducted in accordance with the Ethical Principles in Animal Research adopted by the Brazilian College of Animal Experimentation (COBEA), and the protocol/experiments have been approved by the Ethics Committee for Animal Experimentation of Instituto Butantan (CEUAIB Protocol number 1777050816).

## Author Contributions

LM, MA and SV-A conceived the project. LM and SV-A designed the experiments and wrote the paper. LM and DM-V performed the *in silico* analyses. MA, GS, RR, and GO performed the wet lab experiments and analyses. LM and SV-A analyzed and interpreted the data. DP contributed with informatic resources.

## Funding

This work was supported by the Fundação de Amparo à Pesquisa do Estado de São Paulo (FAPESP) grant numbers 2014/03620-2 and 2018/23693-5 to SV-A. LM, GS and RR received FAPESP fellowships (grant numbers 2018/19591-2, 2018/24015-0 and 2017/22379-2, respectively) and DM-V received a fellowship from Conselho Nacional de Desenvolvimento Científico e Tecnológico (CNPq). SV-A laboratory was also supported by institutional funds from Fundação Butantan and received an established investigator fellowship award from CNPq, Brasil.

## Conflict of Interest Statement

The authors declare that the research was conducted in the absence of any commercial or financial relationships that could be construed as a potential conflict of interest.
