# Supplementary material for: Weighted Gene Co-Expression Analyses Point to Long Non-Coding RNA Hub Genes at Different Schistosoma mansoni Life-Cycle Stages
Source: Front Genet. 2019 Sep 12;10:823. doi: 10.3389/fgene.2019.00823 (PMC6752179; doi:10.3389/fgene.2019.00823)
Supplement: Supplementary file 9 [file DataSheet_1.pdf]

Supplementary Material

1. Supplementary Figures

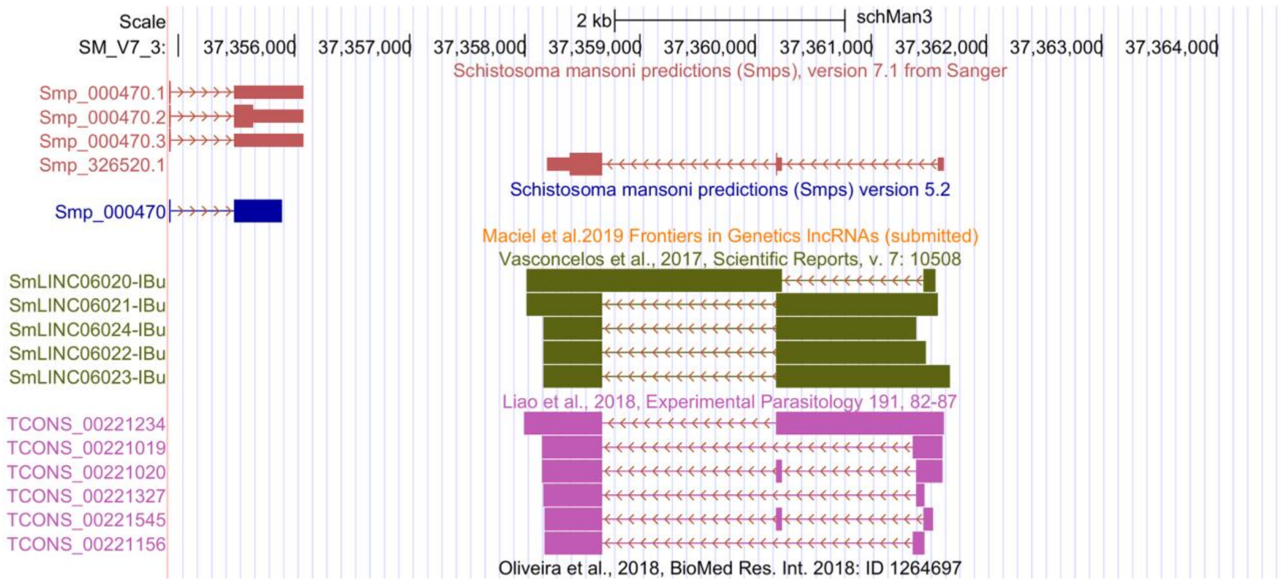

Supplementary Figure S1. Previously annotated *S. mansoni* lncRNAs are in fact partially processed pre-mRNAs in a genomic *locus* encoding a new protein-coding gene. Snapshot of a *S. mansoni* genome browser image, showing a region spanning 9.7 kb on chromosome 3 with coordinates SM_V7_3:37,354,920-37,364,636 (top black row). The red track (top) shows a novel protein-coding gene Smp_326520.1 newly annotated in transcriptome version 7.1, and not present in the old transcriptome version 5.2 (blue track, middle). The orange track (just below the blue track) represents the transcripts annotated in the present work, and it no longer shows any lncRNA in this *locus*. Partially processed pre-mRNAs with intron retention can be recognized among the five supposedly intergenic lncRNAs (SmLINC06020-IBu to SmLINC06024-IBu, grey track) that were annotated in the previous work by Vasconcelos et al. (2017), and among the six different supposedly intergenic lncRNAs (TCONS_00221xxx, pink track) that were annotated in the previous work by Liao et al. (2018). No lncRNAs were annotated in this *locus* by Oliveira et al. (2018) (empty black track at the bottom).

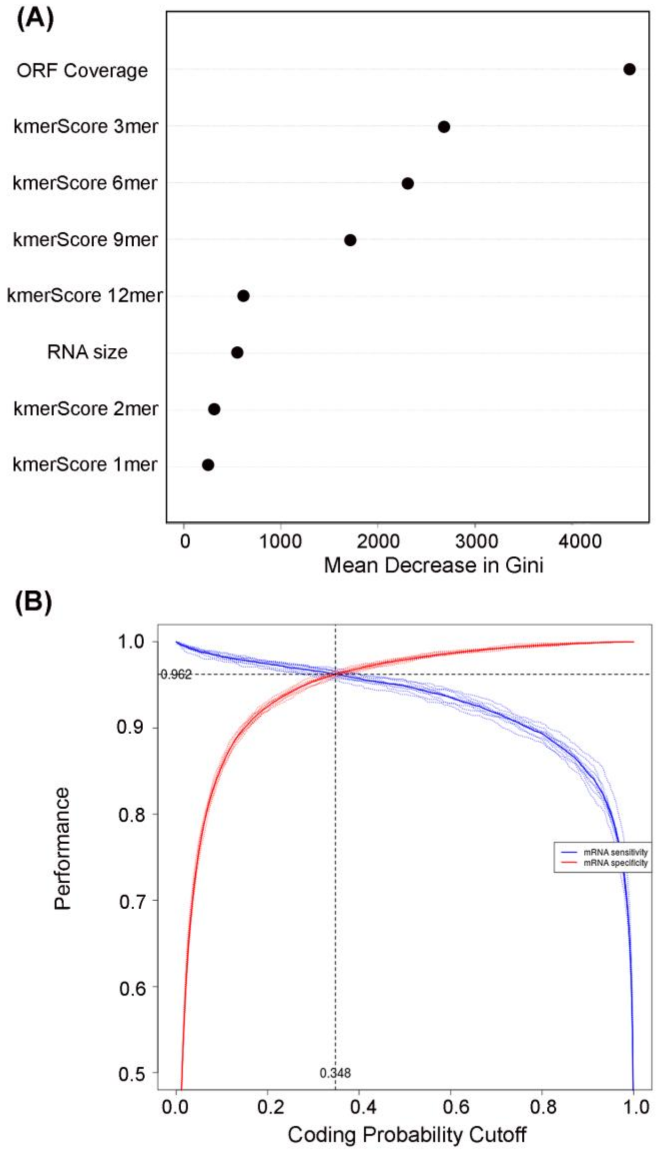

Supplementary Figure S2. Coding probability classification parameters determined by the FEELnc lncRNA classification tool. (A) The graph shows the rank of the eight mRNA sequence features (y-axis) that were used by the Random Forest machine learning FEELnc classifier algorithm to discriminate between known *S. mansoni* mRNAs and putative lncRNAs; these features were ranked based on their discriminatory potential given by the Mean Decrease in Gini metric (x-axis), which is a measure of each feature importance for sequence classification across all of the trees that make up the forest; a higher Mean Decrease in Gini indicates higher feature importance. The most important feature for transcripts classification was the ORF coverage, i.e. the fraction of the total length of the transcript that is occupied by the longest predicted ORF. (**B**) an optimal coding probability cutoff (0.348) was identified, which resulted in 0.962 sensitivity (blue) and specificity (red) of mRNA classification.

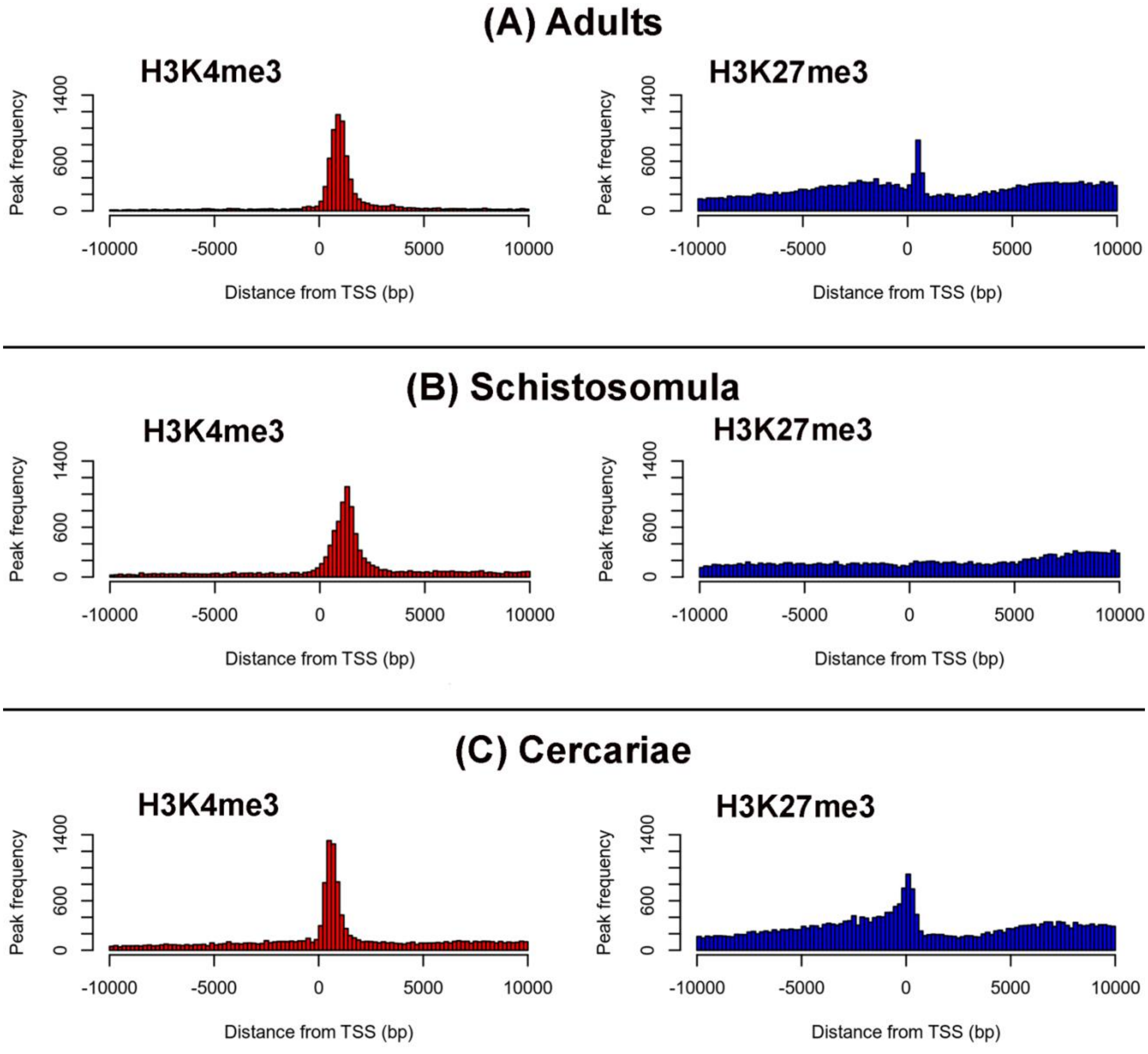

Supplementary Figure S3: Epigenetic histone marks H3K4me3 and H3K27me3 surrounding the TSS of *S. mansoni* protein-coding genes. The frequency of the H3K4me3 marks (red) or of the H3K27me3 marks (blue) mapping within 10 kb around the TSS of all protein-coding genes in (A) adults, (B) schistosomula and (C) cercariae was computed.

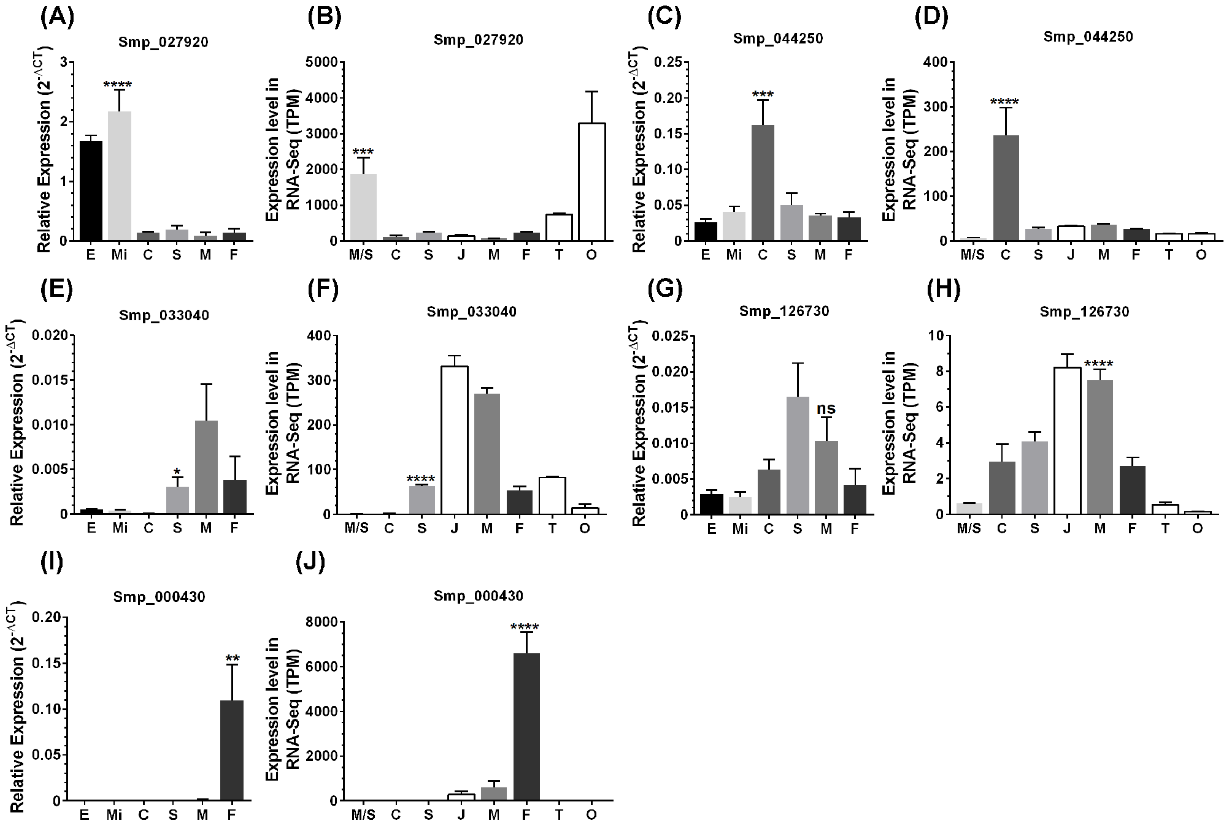

Supplementary Figure S4: RT-qPCR and RNA-Seq (TPM) gene expression level of protein-coding genes used as sample markers. Expression of five protein-coding genes was measured at different developmental stages of *S. mansoni* by the RT-qPCR assay, and their stage-specific expression pattern was compared with their expression as determined by the RNA-seq data. In the y-axis, lincRNAs expression levels measured by RT-qPCR (**panels A, C, E, G and I**) or determined from the RNA-seq (TPM) analysis (**panels B, D, F, H and J**) are shown at the stages indicated in the x-axis, as follows: eggs (E), miracidia (Mi), miracidia/sporocysts (M/S), cercariae (C), *in vitro* mechanically transformed schistosomula cultivated for 24 h (S), juveniles (J), adult males (M), adult females (F), and their gonads, namely testes (T) and ovaries (O). The protein-coding genes relative gene expression by RT-qPCR was calculated against the geometric mean of two housekeeping genes: Smp_090920 and Smp_062630. For each lincRNA, the RT-qPCR expression and the RNA-Seq expression results are shown in two panels side by side, as follows: **(A)** and **(B)** Smp_027920 (Tubulin), a gene that marks miracidia; **(C)** and **(D)** Smp_044250 (Metalloprotease), a gene that marks cercariae; **(E)** and **(F)** Smp_033040 (Lactate dehydrogenase), a gene that marks schistosomula; **(G)** and **(H)** Smp_126730 (5-HTR), a gene that marks adult males; and **(I)** and **(J)** Smp_000430 (Egg Shell Protein), a gene that marks adult females. Bars represent standard deviation of the mean from two to thirty biological replicates for each stage. The ANOVA Tukey test was used to calculate the statistical significance of the expression differences among the parasite stage samples (ns: p-value ≥ 0.05; *p-value ≤ 0.05; **p-value ≤ 0.01; **p-value ≤ 0.001; ****p-value ≤ 0.0001). For clarity purposes, we show only the highest p-value obtained from the ANOVA Tukey test for expression comparisons against one another among the stages.

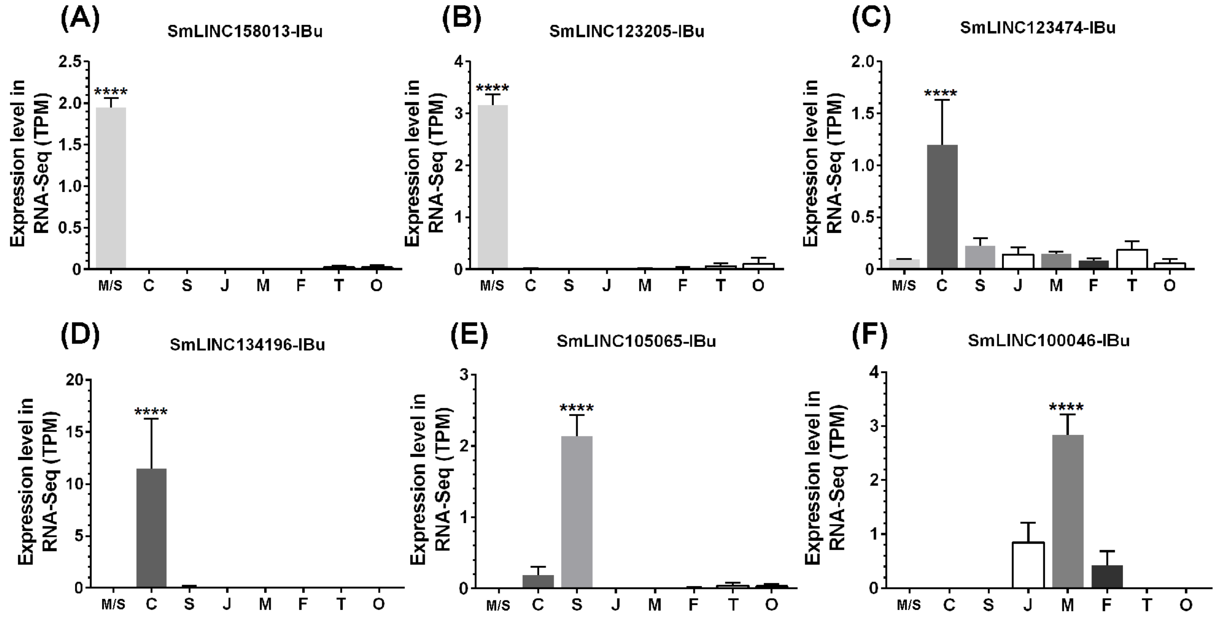

Supplementary Figure S5. RNA-seq expression levels (in transcripts per million, TPM) of the module-specific lincRNAs that had their expression confirmed by RT-qPCR. The six lincRNAs whose gene IDs are indicated at the top of each panel were selected according to their expression levels significantly higher at the given developmental stage of *S. mansoni* indicated in the x-axis, as determined by analyses of the publicly available RNA-seq libraries. The y-axis shows the lincRNA expression level in the RNA-seq assays (TPM) as determined at the stage indicated in the x-axis as follows: miracidia/sporocysts (M/S), cercariae (C), schistosomula (S), juveniles (J), adult males, (M), adult females (F), and their gonads, namely testes (T) and ovaries (O). **(A)** and **(B)** show SmLINC158013-IBu and SmLINC123205-IBu representing the **purple** module, specific for miracidia/sporocysts. **(C)** and **(D)** show SmLINC123474-IBu and SmLINC134196-IBu representing the cercariae-specific **tan** module. **(E)** shows the schistosomula-specific lincRNA SmLINC105065-IBu from the **magenta** module, and **(F)** the adult male-specific lincRNA SmLINC100046-IBu from the **turquoise** module. Bars represent standard deviation of the mean from two to thirty biological replicates for each stage. The ANOVA Tukey test was used to calculate the statistical significance of the expression differences among the parasite stage samples (****p-value ≤ 0.0001). For clarity purposes, we show only the highest p-value obtained in the ANOVA Tukey test for expression comparisons against one another among the stages.

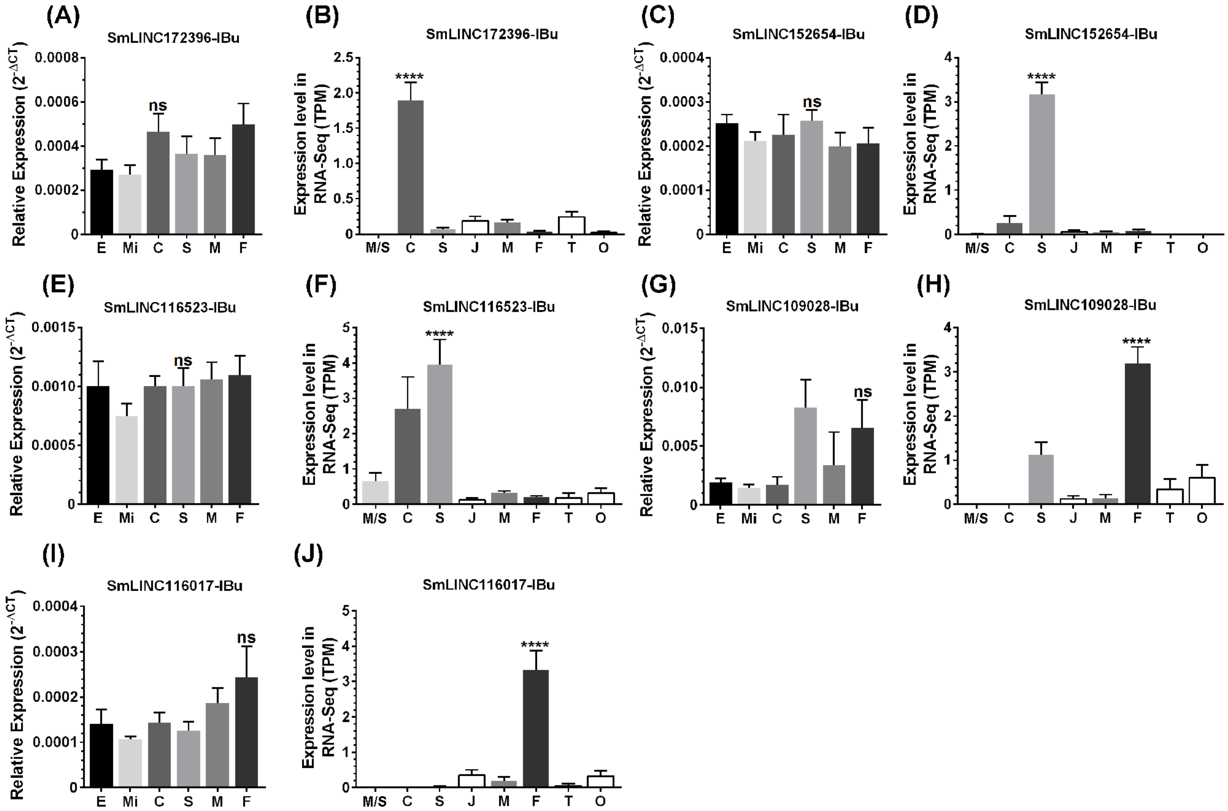

Supplementary Figure S6. RT-qPCR and RNA-Seq (TPM) expression levels of the five lincRNAs that were detected as expressed but did not have the module-specific expression confirmed by the RT-qPCR analysis. Expression of five lincRNAs was measured by RT-qPCR in the RNA from different developmental stages of *S. mansoni* and were not validated as most highly expressed in the same stage as determined from the RNA-Seq data. LincRNAs expression level in the RT-qPCR assay (**panels A, C, E, G and I**) and in RNA-Seq analysis (TPM) (**panels B, D, F, H and J**) was measured in the stages, as indicated in the x-axis: eggs (E), miracidia (Mi), miracidia/sporocysts (M/S), cercariae (C), schitosomula (S), juveniles (J), adult males (M), adult females (F), and their gonads, namely testes (T) and ovaries (O). The lincRNAs RT-qPCR relative gene expression was calculated against the geometric mean of two housekeeping genes: Smp_090920 and Smp_062630. For each lincRNA, the RT-qPCR expression and the RNA-Seq expression results are shown in two panels side by side, as follows: **(A)** and **(B)** SmLINC172396-IBu represents the **tan** module (cercariae); **(C)** and **(D)** SmLINC152654-IBu and **(E)** and **(F)** SmLINC116523-IBu represent the **magenta** module (schitosomula); **(G)** and **(H)** SmLINC109028-IBu and **(I)** and **(J)** SmLINC116017-IBu represent the **pink** module (adult females). Bars represent standard deviation of the mean from two to thirty biological replicates for each stage. The ANOVA Tukey test was used to calculate the statistical significance of the expression differences among the parasite stage samples (ns: p-value ≥ 0.05; ****p-value ≤ 0.0001). For clarity purposes, we show only the highest p-value obtained in the ANOVA Tukey test for expression comparisons against one another among the stages.

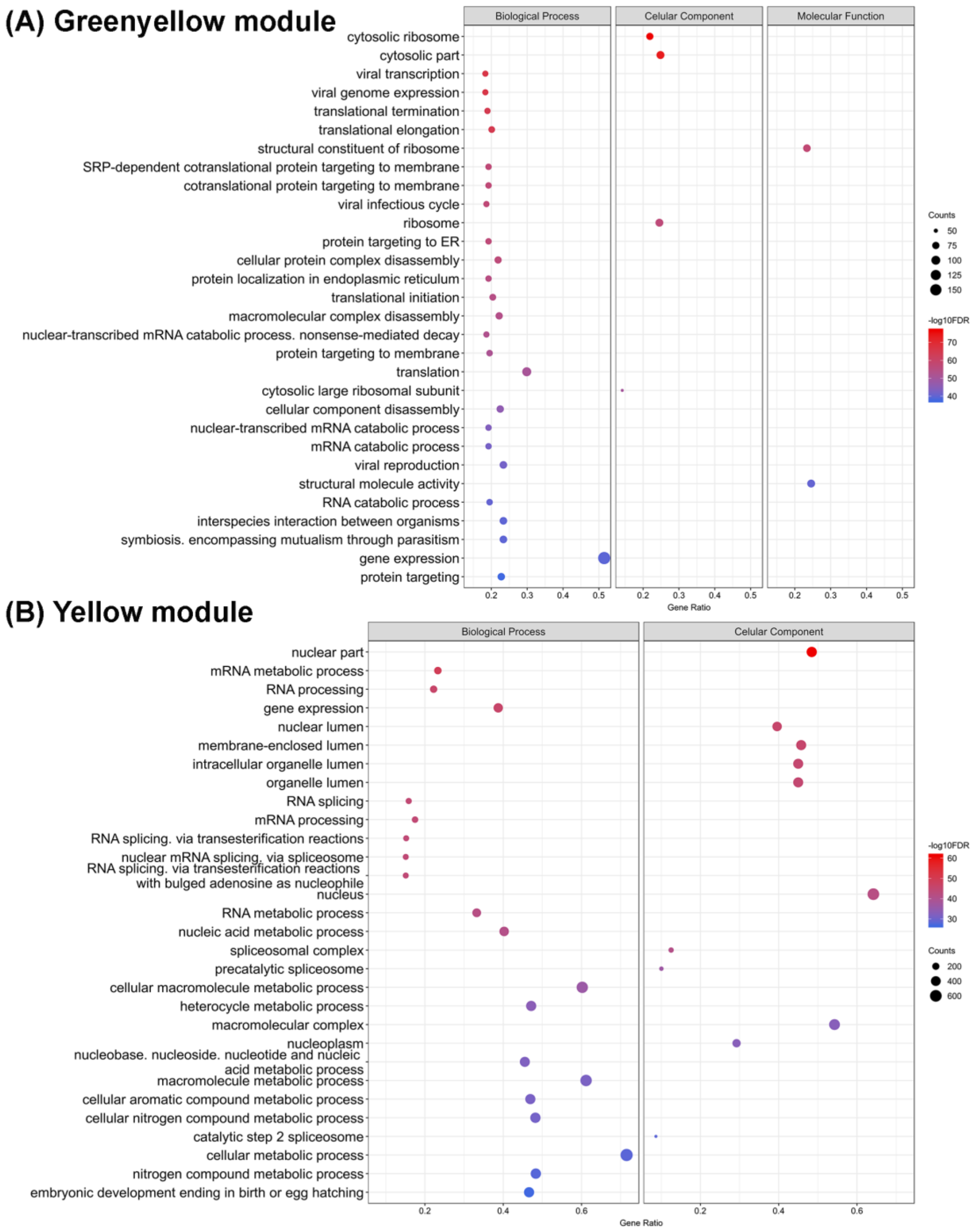

Supplementary Figure S7. Top 30 Gene Ontology most significantly enriched terms for protein-coding genes belonging to the greenyellow and yellow co-expression network modules. At left are the enriched GO term annotations. For the (**A**) greenyellow (gonads) and the (**B**) yellow (females) modules, the enriched GOs are separately represented into the three major GO term categories, namely Biological Process, Cellular Component and Molecular Function. No Molecular Function term was significantly enriched in the yellow module. The size of the circles is proportional to the number of genes (counts scale on the right) in each significantly enriched GO category, and the colors show the statistical significance of the enrichment, as indicated by the -log10 FDR values (color-coded scales at the right).

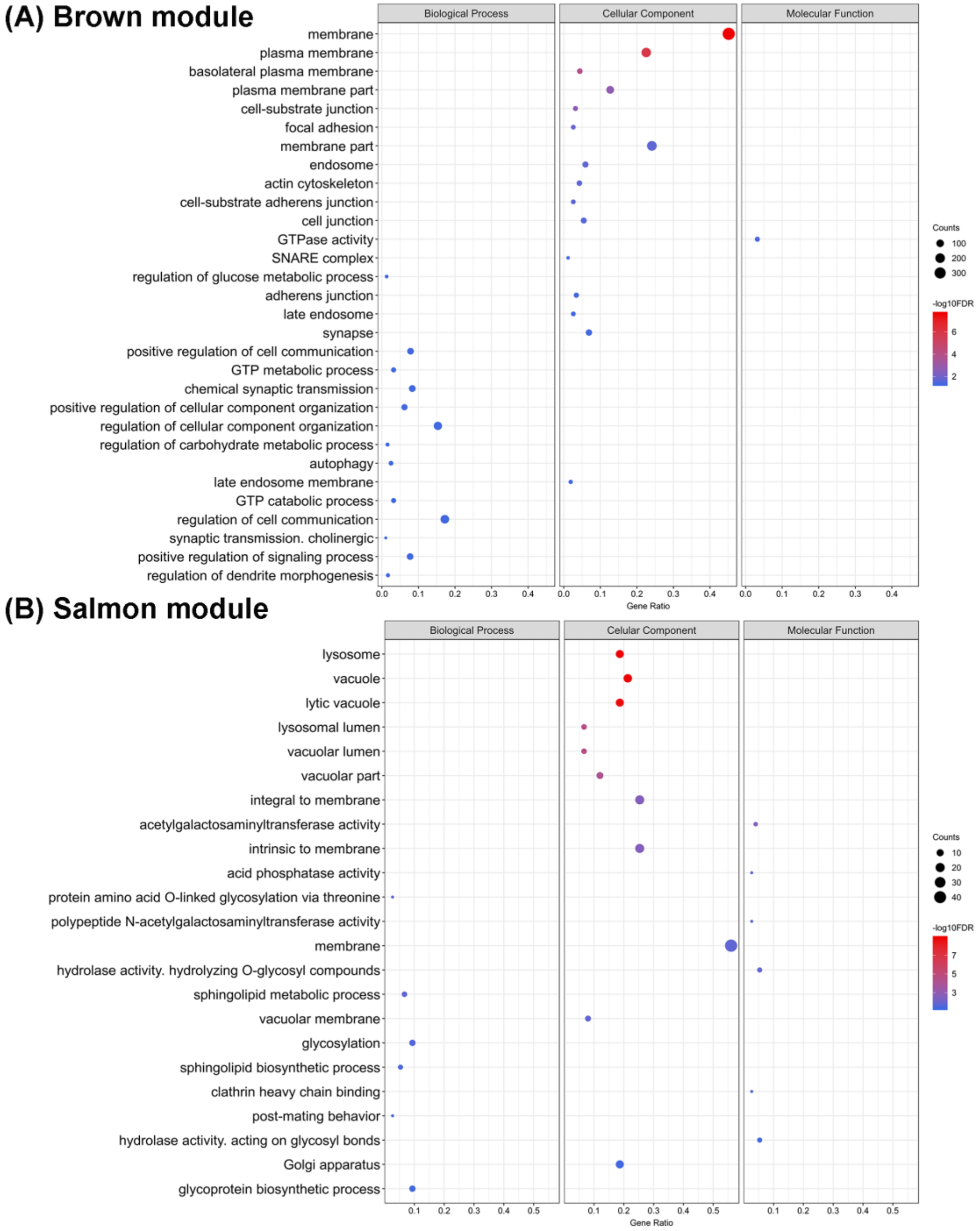

Supplementary Figure S8. Top 30 Gene Ontology most significantly enriched terms for protein-coding genes belonging to the brown and salmon co-expression network modules. At left are the enriched GO term annotations. For the (**A**) brown (gonads) and the (**B**) salmon (gonads) modules, the enriched GOs are separately represented into the three major GO term categories, namely Biological Process, Cellular Component and Molecular Function. The size of the circles is proportional to the number of genes (counts scale on the right) in each significantly enriched GO category, and the colors show the statistical significance of the enrichment, as indicated by the -log10 FDR values (color-coded scales at the right).

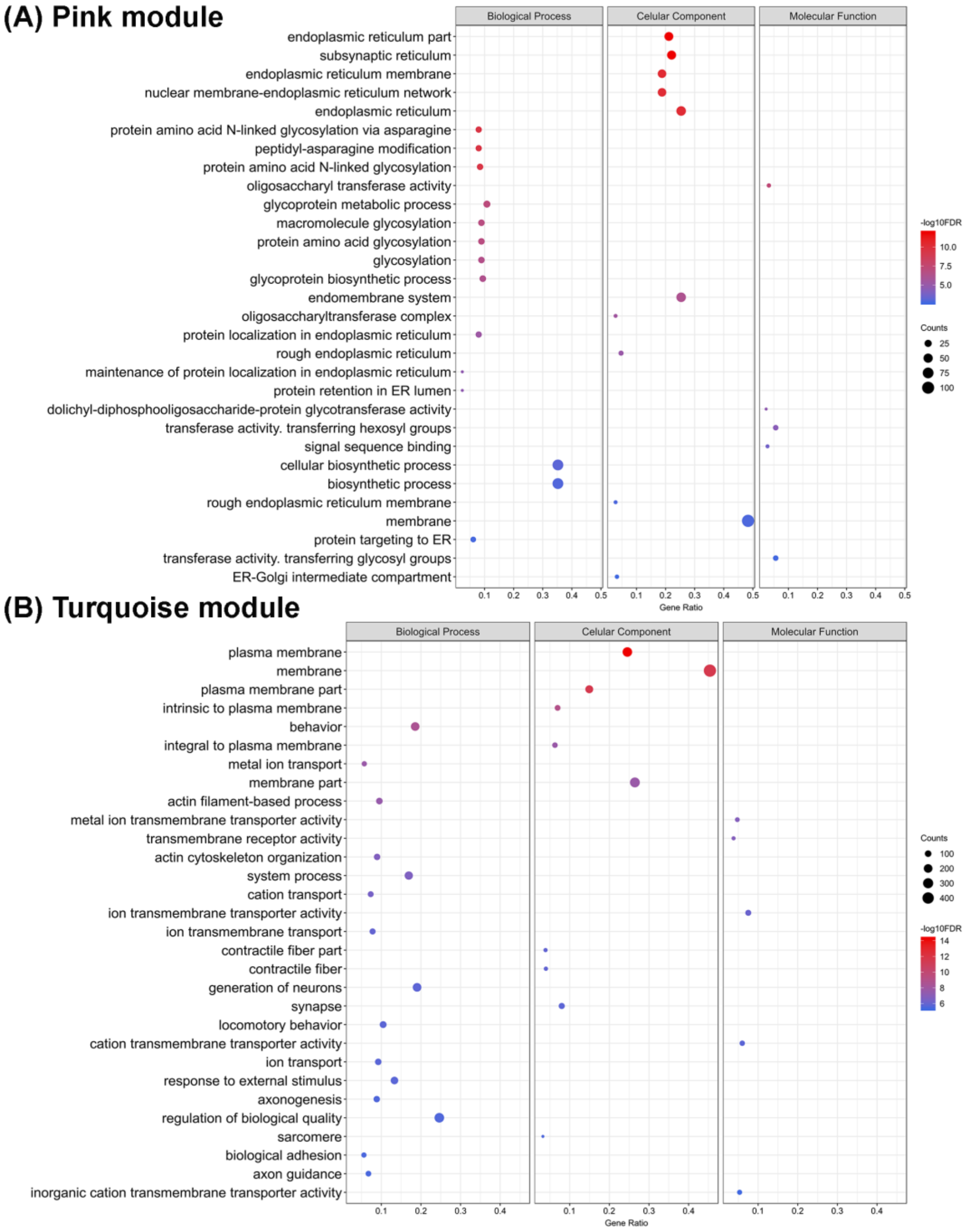

Supplementary Figure S9. Top 30 Gene Ontology most significantly enriched terms for protein-coding genes belonging to the pink and turquoise co-expression network modules. At left are the enriched GO term annotations. For the (**A**) pink (females) and the (**B**) turquoise (males) modules, the enriched GOs are separately represented into the three major GO term categories, namely Biological Process, Cellular Component and Molecular Function. The size of the circles is proportional to the number of genes (counts scale on the right) in each significantly enriched GO category, and the colors show the statistical significance of the enrichment, as indicated by the -log10 FDR values (color-coded scales at the right).

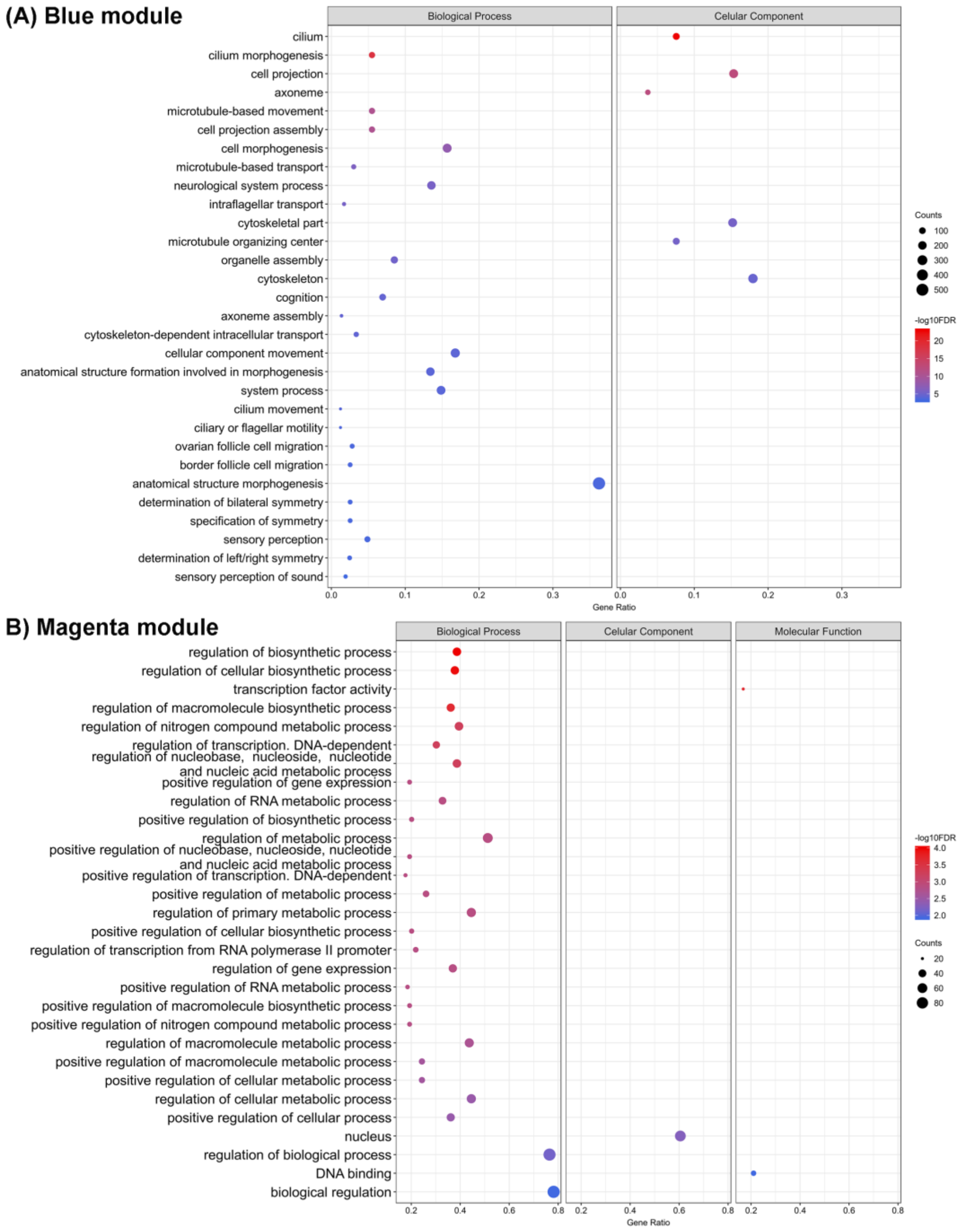

Supplementary Figure S10. Top 30 Gene Ontology most significantly enriched terms for protein-coding genes belonging to the blue and magenta co-expression network modules. At left are the enriched GO term annotations. For the (**A**) blue (females) and the (**B**) magenta (schistosomula) modules, the enriched GOs are separately represented into the three major GO term categories, namely Biological Process, Cellular Component and Molecular Function. No Molecular Function term was significantly enriched in the blue module. The size of the circles is proportional to the number of genes (counts scale on the right) in each significantly enriched GO category, and the colors show the statistical significance of the enrichment, as indicated by the -log10 FDR values (color-coded scales at the right).
